# Moving beyond narrow definitions of gene drive: Diverse perspectives and frames enable substantive dialogue among science and humanities teachers in the United States and United Kingdom

**DOI:** 10.1177/09636625221148697

**Published:** 2023-02-06

**Authors:** Sarah Hartley, Aleksandra Stelmach, Jason A. Delborne, S. Kathleen Barnhill-Dilling

**Affiliations:** University of Exeter, UK; North Carolina State University, USA

**Keywords:** definitions, focus groups, framing, gene drive, public debate, science communication

## Abstract

Gene drive is an emerging biotechnology with applications in global health, conservation and agriculture. Scientists are preparing for field trials, triggering debate about when and how to release gene-drive organisms. These decisions depend on public understandings of gene drive, which are shaped by language. While some studies on gene drive communication assume the need to persuade publics of expert definitions of gene drive, we highlight the importance of meaning-making in communication and engagement. We conducted focus groups with humanities and science teachers in the United Kingdom and United States to explore how different media framings stimulated discussions of gene drive. We found diversity in the value of these framings for public debate. Interestingly, the definition favoured by gene drive scientists was the least popular among participants. Rather than carefully curating language, we need opportunities for publics to make sense and negotiate the meanings of a technology on their own terms.

## 1. Introduction

Gene drive is an emerging biotechnology that enables genetic changes to be spread through a population over successive generations. Scientists can alter how genes are inherited in organisms so that a gene is passed to all offspring. This means it may be possible to design a gene drive modification that could reduce the size of an animal population or even remove a species from the planet (Webber et al., 2015). Potential applications of gene drive are being developed in global health, conservation and agriculture. No gene drive organisms have been released, but some are on the cusp of field trials. This has triggered significant activity in the governance and regulatory frameworks for assessing and managing releases, and interest in whether communities, stakeholders and publics might support such solutions and how they might shape both the technology and its governance (George et al., 2019; Hartley et al., 2021; Long et al., 2020; National Academies of Sciences Engineering Medicine (NASEM), 2016).

Language shapes the understandings of emerging technologies, and there has been a flurry of interest in the language emerging to talk about gene drive (Evans and Palmer, 2018; MacDonald et al., 2021; Nerlich and Stelmach, 2022; Stelmach et al., 2022; Verma et al., 2021). This interest may be a response to the legacy of previous genetic modification (GM) technologies, where language played a powerful role in shaping debates about the desirability and acceptability of these technologies (Doxzen and Henderson, 2020). However, it may also be a result of the gene drive community’s open commitment to responsibility, particularly through community, stakeholder and pubic engagement activities, to inform the technology’s development and secure community consent for field trials (Annas et al., 2021; Emerson et al., 2017; Long et al., 2020; Thizy et al., 2019). Much of the work around language has been driven by Target Malaria’s engagement activities in Africa in support of the development of gene drive mosquitoes for malaria control. Here, Target Malaria has worked closely with communities to find innovative ways to communicate and engage others in their research (Hartley et al., 2019).

Language is a crucial facet of engagement with science and technology, especially at an early stage of field development, when meanings are in flux and scientists and stakeholders are trying to make sense of the technology (Nerlich and McLeod, 2016). Emerging technologies are communicated and understood through frames that help situate new concepts within older and more familiar technologies or experiences and guide their interpretation (Scheufele, 2006). Recent scholarship on the media coverage of gene drive highlights the importance of these frames which draw on a variety of cultural narratives and understandings (Kamenova et al., 2017; Stelmach et al., 2022). Alongside these studies, a different, more prescriptive approach is emerging which tends to advocate a consistent and common definition of gene drive to avoid confusing the general public, improve understanding, and build trust (e.g. Alphey et al., 2020; Cheung et al., 2020; Schairer et al., 2020). Following conversations with gene drive experts, Alphey et al. (2020: 3) suggest the preferred definition of gene drive is a process that ‘promotes or favors the biased inheritance of certain genes from generation to generation’ and a material object described as ‘any genetic element able to bias its inheritance within a population’.

Many of these studies assume rather uncritically the need to persuade publics of expert definitions of gene drive, evaluating different frames in terms of gaining public support or building public trust. Yet governing gene drives involves democratic decisions about the potential future use, regulation and risk management of gene drives, which will require meaningful and culturally relevant dialogue among stakeholders and with wider publics.

Rather than focusing on the transfer of scientific facts from experts to publics, an approach is needed that seeks to understand ‘how society talks about science’ (Bucchi and Trench, 2014), how shared meanings are formed and what cultural resources are drawn upon to make sense of emerging technologies. Such an approach must emphasise the value of communicating science without being guided by fixed agendas or interests, focusing instead on social dialogue (Davies et al., 2009). For example, Bucchi and Trench (2021) argue for rethinking of the purpose of science communication. In their view, many of the current approaches to science communication which emphasise standards and recipes on how to communicate are ‘paternalistic in their mild form, disciplining or engineering the audience in their strong version’ (Bucchi and Trench, 2021: 4) and should move to more inclusive and non-prescriptive formats which would ‘stimulate publics to think about, respond to and discuss science and its role in society’.

We draw on this approach to science communication, which highlights the processes of meaning-making and emphasises the importance of culture in shaping how scientific and technological advances are discussed and debated in public (Bucchi and Trench, 2021; Davies et al., 2019; Horst and Davies, 2021; Macnaghten et al., 2019). In this study, we aim to explore non-prescriptive ways of talking about an emerging technology by designing focus groups in which framings are used not to fix the meanings of gene drive and impose them on participants, but rather to initiate a reflexive discussion around the questions: what are the assumptions underlying this framing? Who would use it and why? In this way, we do not examine the language of gene drive with the goal of assessing or stimulating public support (or opposition) or assessing public understandings of this technology (Davies et al., 2009). Instead, we are interested in exploring the potential of framings to illuminate various aspects of the technology and to obscure some others, as well as their capacity to stimulate participants to investigate them, challenge or offer alternative interpretations. We are interested in how these framings can guide both understandings and misunderstandings of science, and how they can produce unexpected interpretations. The focus of this study is thus to examine ‘the social conversation around science’ (Bucchi and Trench, 2021), or how people talk about gene drive technology and how they think this technology could be discussed and debated. In doing so, our study explores the processes of meaning-making and how various linguistic and cultural resources, including frames, are drawn upon to make sense of this technology (Macnaghten et al., 2019).

In 2020, we conducted four focus groups with humanities, social studies and science teachers in the United Kingdom and United States to explore the value of different gene drive frames to stimulate discussion of this technology. Unlike previous studies, where focus group participants were provided with information materials developed by researchers (MacDonald et al., 2020; Schairer et al., 2020), we used existing resources on gene drive to identify frames that were already in circulation. We decided to use existing frames not to impose meanings on the participants, but rather to use them as ‘conversation starters’ and to ask the participants to reflect about the meanings they carried. Rather than shaping teachers’ attitudes about the technology, the frames enabled a nuanced discussion about what various frames might mean in a given context to a given audience, and how they could generate discussion about the technology and its governance.

## 2. Focus groups with UK and US teachers

**Figure 1.**
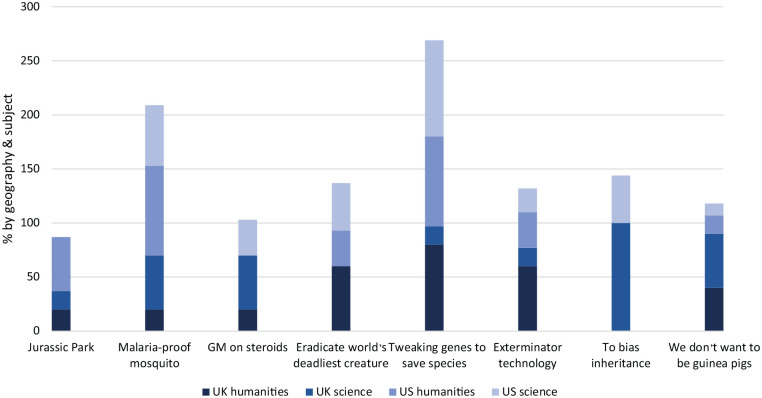
Teacher preference for gene drive frames.

Our study uses focus groups to explore and assess the value of different framings for generating societal debate about gene drive technology (Macnaghten, 2021). This design choice is well-suited to engagement as a form of meaning-making and may even be a way to ‘inject social agency in technological appraisal’ (Macnaghten, 2021: 15). Focus groups involved five design choices:

1. *Context*: People make sense of emerging technologies in a particular context, focus groups should therefore situate the technology in its constitutive domain of practice (Macnaghten, 2021). We asked the teachers to consider the usefulness of eight framings of gene drive for educating their students and for communicating this technology to lay publics. We asked them: (1) What does this framing suggest to you?; (2) Who would use this framing?; (3) Are there any assumptions embedded in this framing?; (4) Would these framings be useful for teaching and discussion? We also conducted a poll in which we asked the teachers to rank their three favourite framings and explain why they chose them (see Figure 1).
2. *Framing*: As technologies are never neutral, but framed for particular purposes, gene drive should be introduced through an inclusive range of frames without closing down or narrowing the issue (Macnaghten, 2021). We selected eight framings that were in circulation in public debate. We sourced them from the sample of media reporting and interviews with stakeholders conducted as part of our other studies on gene drive (Russell et al., 2022; Stelmach et al., 2022). The full description of the rationale and the process of frame selection is included in the Supplemental MaterialAppendi. The frames encouraged either support for or opposition to the technology and focused on explaining the mechanisms of gene drive or the application domain (see Table 1). Framing also featured in our selection of materials provided to participants to ensure some understanding of gene drive. We provided: [1] an open letter from proponents advocating the benefits of gene drive (Target Malaria, 2018); [2] an open letter from opponents calling for a moratorium (ETC Group, n.d.); [3] a hyperlink to a YouTube video entitled ‘What’s a gene drive’ (Risk Bites, 2015); and [4] a hyperlink to a TED talk entitled ‘Gene editing can now change an entire species – forever’ (Kahn, 2016). At the start of each focus group, we showed participants a short video entitled ‘What is gene drives about’ (European Food Safety Authority (EFSA), 2020) and delivered a presentation consisting of 12 slides that outlined the mechanisms of gene drive, potential applications, as well as related political, ethical and societal issues.
3. *Moderation*: The role of the moderator is to keep the group on topic while listening empathetically and ensuring a diversity of voices and to probe differences and convergences between group members (Macnaghten, 2021). The first author moderated the UK focus groups and the third author the USA focus groups. The moderators teased out participants’ reactions to the frames and views on their value for stimulating debate. Moderators encouraged participants to examine frames by drawing on their experience of teaching and communicating complex topics to secondary school students and then reflect on the usefulness of these frames to generate debate about gene drive.
4. *Sampling*: As a qualitative method of research focus groups do not aim to produce statistically representative samples of broader populations, but rather to gain insights from a purposefully selected groups of individuals, drawing on their personal experiences, perceptions and attitudes (Bloor et al., 2001; Nyumba et al., 2018). Focus group participants should be selected based on a balance of shared experience and diversity so that participants feel comfortable interacting, empowered to communicate their perspectives, yet challenged by different perspectives (Macnaghten, 2021). Our focus groups consisted of secondary school teachers based in the United Kingdom and the United States. These countries are at the forefront of gene drive research and gene drive developers have appeared in mainstream media talking about this technology (Stelmach et al., 2022).
  Science literacy is a common goal in both the United Kingdom and United States, resting on the assumption that by the end of high school, or secondary school, people should be literate in science (Liu, 2009). This clearly places responsibility for science literacy with teachers. Teachers, therefore may not be experts in gene drive but are not lay publics. They are experts in communicating science and in explaining complex scientific topics to school children. Science teachers are experienced in communicating biology, while English teachers know how to use language creatively by drawing on framings and stories, and Social Studies teachers are used to communicating about the social, political, and historical contexts of a broad range of issues. In November 2020, we held four online focus groups at 1.5 hours each: 2 with humanities teachers (1 × UK and 1 × US), and 2 with Science teachers (1 × UK and 1 × US). Research was carried out online due to COVID-19-related social distancing restrictions in place at the time. Focus groups were conducted via Zoom. Zoom allowed participants to watch videos and presentations together, and respond to an opinion poll to facilitate discussion.
  In the United Kingdom, focus groups were conducted with five English teachers and six science teachers from state secondary schools in South West England. In the United States, one group was composed of nine secondary science teachers and a second group was composed of six English and Social Studies teachers from North Carolina public schools.
5. *Analysis and interpretation*: Focus group analysis is concerned with what people say and how they say it, in particular uncovering the cultural narratives that enable deliberation and articulation of particular views (Macnaghten, 2021). The role of the analyst is to identify and organise key thematic concerns and discourses, to highlight the cultural resources underpinning these themes and to situate the findings in a relevant theoretical framework (Macnaghten, 2021). We analysed participants’ responses to frames by focussing on what they said about them and how, and also by examining how they assessed their usefulness for communication and teaching. The analysis sought to uncover patterns of sense-makings and points of view, highlighting convergences and differences between focus groups and individual participants. We also sought to uncover mismatches between the intended effects of some frames used in public communication with the actual meanings that were produced and negotiated during focus groups.

**Table 1.** Linguistic expressions/framings used in focus groups discussions.

	Explanatory framings (mechanisms)	Framings of applications and implications of gene drive
Pro-gene drive perspective	*Malaria-proof mosquitoes* *To bias inheritance*	*Tweaking genes to save species* *Eradicate the world’s deadliest creature*
Perspectives critical of gene drive	*Exterminator technology* *GM on steroids*	*Jurassic Park* *We don’t want to be guinea pigs*

GM: genetic modification.

## 3. The meanings of different gene drive frames for public debate

### Jurassic Park

The frame ‘Jurassic Park’ refers to a blockbuster movie about catastrophic consequences of cloned dinosaurs breaking free from a theme park (Watts, 2018).

Associations with Jurassic Park triggered participants’ concerns about control over gene drive. When framed as ‘Jurassic Park’, this technology was described as ‘out of control’ (USH3)^1^ or ‘something which becomes quite uncontrollable and it endangers life’ (UKH2). According to one participant, Jurassic Park offered an apt analogy to gene drive as it conveyed ‘that idea about containing something initially, in a single location [. . .] but then ultimately they lose that control over it’ (USH2). Some participants also thought that ‘control is an illusion that [. . .] scientists have’ (USH3).

For some (UK) participants, the issue of control over gene drive related to concerns over the power of big companies who could ‘just use it for business rather than look at it in a moral way’, an idea that was also ‘very concerning’ (UKH3). As one participant noted, ‘Jurassic Park is driven by the power of business and entertainment [. . .], so it makes you really think about [. . .] the consequences of wanting something to make more money’ (UKH1). Association with business could also raise questions about ‘who controls this once companies get hold of it’, and be used by opponents of gene drive to cast this technology as ‘evil, like the way people view Monsanto’ (UKS2).

The frame ‘Jurassic Park’ also prompted the participants to discuss the potential unintended consequences of this technology (Barnhill-Dilling and Delborne, 2021). They highlighted a potential lack of foresight on the part of gene drive proponents who ‘look at it with rose-tinted glasses and they’re not actually taking into consideration the consequences of their actions’ (UKH3). They also highlighted ‘catastrophic language’ (UKH5) inherent in the ‘Jurassic Park’ frame, and ‘the disastrous feelings associated with it’ (UKS2), conjuring images of ‘impending disaster’ (UKS1), ‘Pandora’s Box’ (UKH6) and of ‘scientists playing with genetics’ (UKS2).

Participants also discussed the ways in which ‘Jurassic Park’ could be used for communicating gene drive to their students. A number of participants (UKH3-5, UKS2,3,5 and USH2) thought that this frame was good for discussion as it brought across ‘the moral, the ethical, the political’ (USH6) issues and it made people think ‘about what does this technology mean’ (UKS5). Participants USS2 and UKH4 had used this frame in their classroom teaching to discuss other issues, while participants USH2, USH4 and USH6 noted the accessibility of this frame and its ‘fun’ aspect that could ‘get attention’ and appeal to children or people with children. By contrast, some participants questioned the appropriateness of this frame, as it misrepresented the feasibility of this technology (USH2) or did not accurately reflect what it can achieve (UKS6, USH2). Participant USH2 in particular was critical of describing ‘something that is so serious [. . .] in the language of a Hollywood movie’, but during the discussion changed their mind and supported using this frame to stimulate ethical debates about science.

### Malaria-proof mosquito

The frame ‘malaria proof mosquito’ refers to gene drive mosquito engineered to prevent the spread of malaria (Neergaard, 2016). For both UK and US participants, this frame denoted positive ideas of reliable solutions to major health problems. When described in this way, gene drive technology sounded ‘very simple, very straightforward and very attractive’ (UKS3), and had positive connotations, because ‘malaria’s bad so if you make it malaria proof then it must be good’ (UKS2). It was also interpreted as safe, because proofing mosquitoes against malaria implied that ‘nothing can go wrong’ (UKH4) and it did not involve eradication of species (USS5), as ‘we’re not talking about destroying any species, the mosquitoes are still there’ (USH6). Proofing mosquitoes against malaria was seen as effective, as ‘it could happen so quickly, it could impact on so many’ (UKH2), and it would remove the need to use antimalarial drugs and insecticides (USS2,3). For two US participants, this even constituted a ‘guarantee’ that mosquitoes won’t be able to make people sick with malaria (USH5), and that the technology is ‘a hundred percent guaranteed, it’s certain and promising and reliable; [. . .] so it has some sort of ethos associated with it’ (USH1).

While acknowledging positive messages behind this frame, a number of participants expressed reservations about its use. They noted that it was ‘emotive’ (UKH5), ‘overly optimistic’ (USH8), ‘selling a dream’ (UKH4) and too futuristic or akin to ‘something from a Marvel or Batman movie’ (USH6 and USH4). Others highlighted the promotional aspect of this framing which offered a ‘positive spin’ on an uncertain technology (UKS1,5) and ‘the best sales pitch’ (USS2) from gene drive supporters. In the words of one participant, the image offered by this frame ‘is obviously skewed to one side and it doesn’t look at any negatives at all’ (UKS1). By contrast, a US participant (USH3) with an experience of living in a country affected by malaria, was the most supportive of using this frame. Despite these limitations, the participants thought that this frame was useful for teaching and communicating, as it conveyed well the potential use of gene drive mosquitoes in malaria prevention.

Unexpectedly, the ‘malaria-proof mosquito’ frame also revealed deeply contentious ethical questions about public health interventions’ impact on population growth. Some UK participants (UKH2-5) speculated about the consequences of using gene drive mosquitoes in developing countries where curbing fatalities could increase the rate of population growth; they further discussed impacts on ‘natural selection’ – presumably referring to evolutionary processes that might eventually protect humans from malaria. The conversation included the need to provide support for growing human populations, which would otherwise be smaller because of malaria, in poorer regions of the world where resources are already scarce. While this line of reasoning created deep discomfort among our facilitators, we report it as a reminder of the proximity of discourses of race, poverty, justice, and development to particular applications of gene drive technology.

### GM on steroids

The frame ‘GM on steroids’ refers to the potential of gene drives to spread quickly through the population (Avaaz, n.d.). For roughly half of UK and US respondents (UKH1-5, UKS2-4,5, USH1,2,4, USS5), it evoked negative connotations, mainly because of its use of two terms perceived to be controversial, namely, ‘steroids’ and ‘GM’. ‘Steroids’ made the participants think of gene drive technology as being ‘something that is completely uncontrolled’, ‘unmonitored’, ‘difficult to stop’, or ‘out of control to the point where you’re unable to draw back’ (UKH1,2,4, UKS6, USH2, USS6). It was also described as ‘having an unfair advantage’ (USH1), ‘abnormal, inherently wrong or altered’ (UKH4) or even being ‘illegal’ (UKH5) due to its association with excessive weight lifting and with doping in sport (UKS4). There were a few exceptions to this view; teachers with personal medical histories of using steroids for health reasons found these interpretations confusing.

For some other participants, the term ‘GM’ posed problems (USH2) as ‘a lot of people won’t know what GM stands for’ (UKH3), and in the US context the term could be read as ‘General Motors’ (USH5) or a ‘general manager of this sport franchise [who was] on steroids’ (USH6). This meant that some UK and US participants found the frame ‘GM on steroids’ hard to understand, as it failed to connect to people’s lived experience (USH2,6, UKH3, UKS2,3). Other participants emphasised ‘the bad rap’, ‘negativity’, ‘the fear factor’ and ‘stigma’ underlying the term GMOs (USH2-4, UKH2,5, UKS2,3,6) and noted that the fear of GM foods could affect perceptions of gene drive.

At the same time, many UK participants identified this framing as ‘sensationalist’, ‘alarmist’, ‘dramatic’, ‘clickbaity’, ‘hyperbolic’, exaggerated to the point of being ‘almost amusing’ or taken from ‘trashy tabloids’ (UKH1,3-5, UKS2,3). Although this frame could attract attention, the participants were divided as to whether it explained or obscured what gene drive was. As some participants noted, it could reinforce pre-existing opinions, given the confusion created by the terms ‘GM’ and ‘steroids’. For some participants, however, the frame was ‘a good discussion point’ (UKS3) as it reflected some of the features of gene drive technology (UKS4,5) and was ‘accurate’ (USS3) and ‘relatable’ (USS1,4) to lay audiences.

### Eradicate the world’s deadliest creature

The frame ‘eradicate the world’s deadliest creature’ was used in the media coverage of gene drive (Berezow, 2016) and by gene drive funders and supporters (CDC, n.d.; Gates, 2014). Across all the focus groups, participants (UKH2,5, UKS1-3,5, USH2,5, USS3) found this frame unclear and thus uninformative about the future target of the gene drive technology. For some, it related to humans, deemed the deadliest creatures (UKH2,5, UKS3), while others thought of parasites (UKS5), spiders (UKS2) or tsetse flies (USS3). Some argued that the frame’s vagueness had value in communication and teaching as it ‘piques curiosity’, is ‘good to grab attention’ and ‘start a lecture’ and to make ‘kids come up with different things’ (USH2,5,6).

It was unclear to participants how effective this frame would be in garnering support for the technology as it ‘narrows the conversation’ (USS5), sounds ‘pushy’ and implies that ‘you would be ridiculous not to want to do this’ (USS1). Some participants stressed that potential public resistance to this type of framing could be driven by an opposition to the inflammatory language. As one US participant explained, ‘I would not trust it at all. [. . . It’s] so much over the top I was immediately turned off; the more extreme you tend to go the more I’m like, no’ (USH2). Some others noted, however, that the frame, though drawing on aggressive words of ‘eradication’ and ‘the world’s deadliest creature’, was ‘accessible’ to lay publics (UKS5), and still ‘positive’ (USS5), especially given that ‘we eradicate different things’ already (USS6). The ethics of eradicating species also came to play in the discussion. Several participants highlighted potential public concern that elimination of mosquitoes could lead to elimination of other species (UKH3-5, UKS3, USS2,9). For some others, however, the perspective of eradicating malaria-carrying mosquitoes made gene drive an ‘exciting technology’ (USS3).

### Tweaking genes to save species

‘Tweaking genes to save species’ refers to gene drive used to eradicate invasive species that threaten the survival of native ones (Okumu, 2021; Rosner, 2016). For the majority of participants, this frame cast gene drive in a positive light, mainly through the use of the word ‘tweaking’ to describe GM. This use also caused surprise, as, in the words of one participant, ‘there’s always this idea that modifying something [. . .] has this connotation of being quite radical’ (UKH1), while GM of animals was here presented as ‘benign’, ‘mild’, ‘gentle’, ‘genteel’, or ‘minor’ (UKS3,6, UKH1,4, USS3, USH1). Other UK participants compared this language to a political discourse of ‘a conservative broadsheet’ (UKH2) or ‘the centrist Guardian’ (UKH5) that sought not to create controversies by being ‘offensive’, while some others described it as ‘sweet’ and ‘a bit Mary Poppins’ or ‘a Father Christmas [. . .] bringing a bit of gene tweaking’ (UKS5,6). Participants also felt that the word ‘tweaking’ ‘is not scientific’ and drew on lay language (USH3, UKS2), which downplayed the significance of modifying genomes of animals. UK humanities teachers noted that this was achieved by some ‘clever phonetics’ and the use of ‘soft sounds’ (UKH3-5) that could make people think more positively about GM, for example as a ‘nice ribbon of DNA going on’ (UKH2).

The participants noted the contrast of the understatement of ‘tweaking genes’ with the exaggeration of the statement of ‘saving species’ which was seen as ‘extreme’, ‘grandiose’, or ‘emotive’ (UKH4, UKS1). When put together, these phrases created a ‘very positive’ image of gene drive technology as ‘you’re tweaking because you’re trying to do something good’ (UKS2), and ‘everybody likes to save something’ (USH5). At the same time the participants identified a persuasive function of the frame which conveyed the potential efficiency of gene drive technology by contrasting ‘just minute amount of change’ and ‘amazing possibilities’ (UKH2), and by emphasising ‘amazing consequences from one little tweak’ (UKH5). In the words of one science teacher, ‘It sounds rather wonderful; we’re just doing a little tiny thing and we’re going to save the world’ (UKS3).

US participants pointed out that rather than eliciting positive reactions, this frame could have an opposite effect. This was because of hearing ‘tweaking’ as an understatement of the significance of GM, which is usually associated with powerful biotechnologies like ‘designer babies’, test tube babies, or GM food (USS3,4,8). According to one participant, ‘[it] makes me a little nervous, just thinking about it. [. . .] just seeing the word tweaking’ (USS7). Another one explained: ‘Tweaking [. . .] makes me think of a doctor who has a giant needle and is like, no, this isn’t really going to hurt. I feel in the general public, if somebody with a lab coat was like, we’re just tweaking things, people are going to go, oh hell no’ (USH2). For some other US participants, this frame was misleading, as ‘there’s a sense of sneakiness with the tweaking’ which creates an impression that people ‘are being duped’ (USH1,4). And finally, participants noted that in the United States, the term ‘tweaking’ can have connotations akin to ‘freaking out’, which prompted negative associations.

In spite of these criticisms, many participants described this frame as ‘positive’, good for teaching and communication, ‘accurate’ about technology, and ‘relatable’ for lay publics (USH1,3,4,6, USS1,2-5,7,9). Despite being seen as promotional by some UK participants (UKS2,3), some US participants appreciated its ‘mild’ and ‘neutral’ language and ‘stay[ing] away from inflammatory language in science’ (USS3,8).

### Exterminator technology

The frame ‘exterminator technology’ refers to gene drive’s potential to eliminate species, and it was used by opponents of this technology speaking to the press (Achenbach, 2018). Participants thought that the frame was ‘emotive’ or ‘emotionally charged’, and it sounded ‘scary’, ‘very worrying’, ‘out of control’, ‘drastic’ and ‘violent’ (UKS1-3, UKH2,3, USH4, USS8). Some argued that this phrasing made them ‘afraid for their kids’ (USH2) and ask ‘who is behind it’ (UKH4), as ‘it’s not something that an individual should be deciding’ (UKH3). Some of these fears were prompted by associations with the Hollywood movie *Terminator* starring Arnold Schwarzenegger as a cyborg assassin (UKH1, UKS2,4,5, USH2,4,5). When framed in this way, gene drive was described as ‘the science fiction thing: that they’re coming for humans’ (USH2, USH5), and a technology that is ‘dehumanising’ (UKH2,3). It also made participants think of ‘war’, ‘death’, ‘total wipe out’, ‘killer technology’, killing of animals and violence (UKH1,3,5, USH5, USS5,8).

For other participants, this frame brought to mind exterminator businesses dealing with domestic pests. According to one teacher, this made the technology look ‘sinister’ because ‘when you call an exterminator you call them in to do something unsavoury and unpleasant, [. . .] and it’s almost a ‘we won’t talk about it afterwards’ (UKS1). It also made it look ‘scary’ as ‘simultaneously we’re thinking about Arnold Schwarzenegger [. . .] and the guy who comes in and sprays the corners of my house’ (USH4). Not all teachers thought these associations made gene drive technology scary. To some, it offered a ‘targeted approach’ which gave ‘a peace of mind’, and felt ‘cool’, ‘positive’, ‘research-based’, and ‘controllable in a good way’ (USS2, USH1,3,6, UKS2).

A number of participants resisted the dichotomy of ‘positive’ and ‘negative’ reactions to this frame, focussing instead on its educational and communication potential. Some found that the association of the words ‘exterminator’ and ‘technology’ was ‘odd’, ‘weird’, ‘foreign’ (UKH2,4, UKS2) and that its message was not clear, shifting between ‘rat poison’ and ‘Ghostbusters’ (UKH2), a reference to a sci-fi comedy about a ghost-catching business in New York City. This and other comments suggested that at least for some participants this type of scare messaging was not taken seriously. UK teachers in particular argued that the hyped message carried by this frame could be exploited by interest groups, such as protest groups, lobbyist, populist politicians, tabloids or social media (UKS1,3, UKH1,4,5).

### To bias inheritance

The term ‘to bias inheritance’ conveys the mechanism by which gene drives operate (Alphey et al., 2020; Kirkpatrick and Light, 2015). For a number of UK and US participants, especially those who taught science, the frame spoke directly to the science of gene drive and was described as ‘neutral’, ‘not loaded’, ‘unloaded’, ‘the least sensational’, ‘probably most accurate’, ‘most scientific’ or ‘a basic scientific term which doesn’t have any inherent positive or negative connotations’ (UKS1-3,5,6, USH2, USS8). Some participants still thought the frame was slightly misleading, as it did not reflect the scale of change in the reproductive patterns introduced by gene drive (UKH5, UKS1).

By contrast, some UK humanities teachers found that rather than referring to science, the frame linked directly to social issues, as the term ‘inheritance’ was ‘loaded with the idea of humanity [. . .] and how we see our own progeny carrying on’ (UKH2-5). As one participant put it, the word ‘inheritance’ denoted ‘the world that we inherit, [. . .] and what we are leaving for the next generation’ (UKH2). This meant that for a number of participants this frame invited an exploration of ethical issues related to reproduction and social justice.

One of the most unexpected associations sparked by this frame was the issue of ‘designer babies’ and deciding about the characteristics of babies to be born in the future (UKH2,3,5, UKS3,6). In the words of one participant the frame of ‘biasing the inheritance’ was about ‘the genetic modification for designer babies’ (UKH3). For some participants, this entailed a possibility of choosing to have a ‘blue-eyed child’ (UKH5) or ‘a child to be faster, naturally before they’re born’ and fit enough to participate in the Olympics (UKH3). While for one teacher the impact of biasing inheritance amounted to ‘eugenics’ or even ‘genocide’ (USH4), others spoke of ‘unfairness’ of favouring something or someone at the expense of something/someone else (USS5,6, UKS1,3, USH3). This could mean favouring a particular gender or being biased towards some ethnic or social groups. For example, the idea of skewing the ratio of males and females in animal populations led one participant to worry about cultures ‘that view one gender above the other’ (UKH3). Another participant interpreted this frame in the context of recent Black Lives Matter movement: ‘if we’re talking big like look at some of the political unrests we’re having with things like certain ethnic groups being treated badly, are we going to see a bit of a . . .; I really worry about the ideology of that actually’ (UKH5).

The discussion revealed a significant difference of opinion about the usefulness of this frame for talking about gene drive – notably divided by academic discipline. While most science teachers thought it was neutral and accessible for lay audiences (UKS3), humanities teachers tended to see this frame as troubling, carrying ‘a strength of feelings’ and denoting social controversies rather than scientific issues (UKH5, USH5).

### We don’t want to be guinea pigs

Finally, the frame ‘we don’t want to be guinea pigs’ was selected from a newspaper article critical of gene drive trials conducted in Africa (Pujol-Mazzini, 2019). As some participants argued, the ‘emotive’, ‘emotional’, ‘alarmist’ and ‘highly charged’ language of this frame directly involves the public into the debate through the use of the pronoun ‘we’ (UKH2,4,5, UKS6). This strategy emphasised the role of ordinary people in the process of developing, testing and deploying of new technologies. Rather than speaking of nature, animals or plants, it focussed on humanity and on the potentially negative consequences of using gene drive for the most disadvantaged groups and in less developed countries. For some, mostly UK participants, this frame drew on ‘a lot of fear’, created ‘that irrational feeling’, and sought to ‘scare people’ about gene drive (UKH4,5, USH1, UKS6). This even amounted to ‘NIMBYism’ (UKH2, USH1) and sparked comparisons with protest slogans, tabloid journalism, or social media campaigns (UKH1,2,4).

By contrast, other participants emphasised justified concerns about new and ‘untested’ technology among lay publics and warned about technologies driven by ‘good intentions . . . [which] come out wrong’ (UKS2, USS4) and which could lead to ‘wrecking the whole ecosystems’ (UKS2, USS4,9).

For some UK and US teachers, the frame harkened back to cruelty of unethical experiments from the past. As one participant noted, the phrase ‘has connotations of pain and death because of associating them with scientific experiments [. . .] so straight away it automatically presents that negative idea that gene modification is actually something that is really deadly’ (UKH3). Other participants noted that being a guinea pig meant being ‘helpless’ (UKH5) and ‘being ‘desperate’ (USH5), and implied ‘a lack of consent’ (UKH5), or even an ‘imposition’ of a technology in other countries which haven’t developed it (UKS1,3). For a few teachers this frame brought to mind ‘drug trials’ (UKS5) or unethical experiments such as ‘the [Tuskegee] syphilis trial in the 1950s [. . .] being done to unwilling communities’ (USH2). These and other participants (UKH1,5 UKS1-3, USH4, USS2,8) thought that the frame expressed the views of people opposing the science who otherwise would not have a say about scientific matters, such as marginalised sections of society or populations of poorer countries. As one participant put it, ‘this is the rural farmers in less developed countries holding placards against big biotech multinationals, isn’t it? [. . .] And not having the autonomy to control what’s going on in their area’ (UKS1).

This led some participants to discuss the question of how science could progress without being harmful or unethical (USH3-5) and how to make it more democratic. In the words of one participant, it was important to make sure that ‘there’s an equal voice in whether this is used across all parties’, and that ‘people from all different sectors of society are understanding what’s happening and how this could potentially affect people’ (USH4).

#### Cross-group comparisons

Teachers were also asked which framings they would be most likely to use for teaching and communicating gene drive (Figure 1). The focus groups revealed that professional divides between humanities and science teachers influenced their preferences for certain frames more than the country where they lived. The difference was most striking during the discussion of ‘to bias inheritance’ frame, where none of the humanities teachers endorsed it in the poll, while it was chosen by over a half of science teachers. Similarly, the frame ‘GM on steroids’ garnered much less support among the humanities teachers who indicated that the scientific terms ‘GM’ and ‘steroids’ could be unclear or misleading for the general public. By contrast, the humanities teachers expressed a much stronger support for the use of the frame ‘Jurassic Park’ than science teachers. They were also more likely to make references to popular culture topics, such as comedies or science fiction movies, during the discussion.

At the other end of the spectrum, both groups expressed similar levels of support for some other frames – for example, a relatively high support for the frame of ‘malaria proof mosquito’, which was considered useful for communication, and a moderate endorsement for the frame of ‘we don’t want to be guinea pigs’, which by some participants was considered controversial but still useful for the debate. Overall, humanities teachers were more likely to assess a frame in terms of its potential for starting a discussion, while science teachers were more likely to emphasise its accuracy in reflecting various aspects of the technology.

Where the participants lived played an important role in shaping their preferences for particular framings. This was evident for example, during the discussion of the frame ‘malaria-proof mosquito’, which elicited almost twice as much support among the US than the UK teachers. While both groups found this frame useful in conveying the potential of gene drive technology, the UK participants found it too promotional and optimistic in emphasising its benefits. The discussion also revealed a difference in the support for the frame of ‘tweaking genes to save species’ which we attribute to national differences in slang terms.

We found that cultural and historical context shaped interpretations of frames. For example, US participants were less likely to support the frame ‘we don’t want to be guinea pigs’ given the history of unethical medical experiments conducted on disadvantaged groups. By contrast, UK participants were more likely to endorse it as in their view it could potentially convey the point of view of social groups deprived of a voice. In general, UK participants were more likely to express concern about the power of big business in the context of emerging technologies. They were also more likely to associate some linguistic expressions with certain journalistic styles reflecting political stance of tabloids, liberal or conservative newspapers, or expressing the point of view of particular stakeholders (technology developers, NGOs and protest groups, etc.).

## 4. Discussion: The value of gene drive frames

This study explored how science and humanities teachers responded to various frames of gene drive and reflected on their usefulness for discussing the technology. We found the frames’ meanings were never fixed, and that participants were able to question them and negotiate their meanings by situating them in the context of their expertise and experience. Throughout the discussion, new meanings emerged, often revealing mismatches between the intended effects of frames (e.g. to support or to critique gene drive technology) and their actual interpretation by the participants.

The aim of public communication is to start the social conversation around science which is akin to an interactive, open-ended and unpredictable communication (Bucchi and Trench, 2021). While such conversations can accommodate different formats and be more or less structured, they invariably draw on culturally specific images and ideas about science, prioritising the topics of interest to the public. Rather than simply accepting the frames as carrying the ‘truth’ about gene drive, the participants were willing to use them in the discussion to emphasise various aspects of the technology, or to deploy them as a counter-argument to opposing claims. In general, discussions of the frames revealed high levels of awareness and sophisticated understandings about the ways in which different frames could be used rhetorically by various stakeholders to highlight risks and benefits, or to garner support or stir an opposition to gene drive. Even when participants disagreed with the utility of the discussed frames, these frames still allowed them to explore both the technical information about gene drive applications and the societal issues surrounding this technology.

Frames triggered different meanings and interpretations dependent on the context of time and space as well as on the lived experiences of the participants, and those meanings were difficult to control. For example, frames deemed to be ‘neutral’ and convey scientific facts could actually have a polarising effect (see also MacDonald et al., 2020) because of the context in which they were deployed. This was most evident in the case of the frame ‘to bias inheritance’ which created major negative connotations among the participants, especially for those who saw ‘bias’ not as a specialist term, but as a word denoting a threat to fairness and justice. The non-scientists in our focus groups voted this frame their least favourite out of the eight descriptive frames. This result might be considered surprising given that the frame is the preferred choice of the gene drive community (Alphey et al., 2020; NASEM, 2016). However, the participants interpreted this frame in a certain historical moment where debates and social protests about social and racial injustices were taking place in the United States and United Kingdom. This shows that there will always be complex and sometimes unpredictable contexts in which people make sense of technologies, and that it is impossible to fix their meaning once and for all by choosing a frame deemed ‘neutral’ or appropriate.

Our research has important implications for scientists and communicators keen to shape lay understandings of an emerging technology through a careful frame selection (Nisbet and Mooney, 2007) or an emphasis on the language that should, or should not be used. Alphey et al. (2020) claim that common language and standardised definitions will improve communication with stakeholders and publics. They argue the lack of common terminology could confuse the public and lead to the technology’s rejection. Our research suggests that communicating about gene drive is not qualitatively different from communicating about other technologies; stakeholders and publics are keen and able to understand the science sufficiently to discuss its implications with thoughtful attention to risks, benefits and trade-offs (Farooque et al., 2019; Hartley et al., 2019; Jones et al., 2019). Importantly, our study reveals a mismatch between the desired and actual effects of some communication strategies (Davies et al., 2019). It shows that the strategy of selecting frames and choosing what should or should not be said about a technology could backfire, as frames that might be seen by many as generally positive often carry undertones of scepticism and worry. Our study provides a reminder that publics interpret messages in many nuanced ways and their adoption or rejection of some framings can stem from different reasons than those anticipated by science communicators (Hall, 1973; Hall et al., 2003). It also suggests that deliberations where publics can arrive at their own or shared interpretations can enhance more nuanced understandings of emerging and controversial technologies. This would require a more open-minded approach to communicating, where messages about science are underpinned by the desire to share knowledge and co-produce language to talk about it, rather than by the focus on promoting its applications (see Weingart and Joubert, 2019). This would also require the gene drive community to open up to the possibility that communicating about the technology should be guided by publics’ interests and concerns and be more inclusive and responsible, something that developers have already committed to in public (Long et al., 2020; Russell et al., 2022).

Stelmach et al. (2022) have shown that scientists in the field of gene drive have acquired a new ‘moral authority’ through media reporting of their responsibility, expanding their scientific and expert authority and developing a new and closer relationship between science and society. However, our research suggests that this moral authority should be treated with caution. The void we have identified between the language that scientists prefer and the language publics prefer means that scientists cannot and, perhaps, should not be the primary developers of a language for gene drive. The desire to build public trust is often driven by a desire to gain public acceptance through educating and informing publics, and hides important political questions about how technology is owned and controlled (Wynne, 2006). Efforts to build trust and improve communication could backfire unless they are supported by robust social science research that engages with diverse publics and stakeholders and reflects on these political questions. In order to meaningfully build trust, we need to do more than convene scientific experts to agree on common terms and, instead, understand how language is used and understood by the diversity of actors who will participate in making decisions about gene drive development and deployment. The details of the technology must certainly be part of the conversation, but communicating with broader publics to build trust will require just as much careful attention to values, issues of power and control, and the ways that risks and potential benefits will be distributed and managed (Bucchi and Trench, 2021). Rather than focussing on attempts to fix the meaning of a technology or guide public understanding of it through carefully curated language, we need spaces and opportunities for debates where publics can make sense and negotiate the meanings of a technology on their own terms.

## Supplemental Material

sj-docx-1-pus-10.1177_09636625221148697 – Supplemental material for Moving beyond narrow definitions of gene drive: Diverse perspectives and frames enable substantive dialogue among science and humanities teachers in the United States and United Kingdom Supplemental material, sj-docx-1-pus-10.1177_09636625221148697 for Moving beyond narrow definitions of gene drive: Diverse perspectives and frames enable substantive dialogue among science and humanities teachers in the United States and United Kingdom by Sarah Hartley, Aleksandra Stelmach, Jason A. Delborne and S. Kathleen Barnhill-Dilling in Public Understanding of Science

## Notes

1. We are using the following annotations to describe our research participants: UKH–UK humanities teachers, UKS–UK science teachers, USH–US humanities and social sciences teachers, USS–US science teachers.
