# Supplementary material for: Moving beyond narrow definitions of gene drive: Diverse perspectives and frames enable substantive dialogue among science and humanities teachers in the United States and United Kingdom
Source: Public Underst Sci. 2023 Feb 6;32(6):727–44. doi: 10.1177/09636625221148697 (PMC10363919; doi:10.1177/09636625221148697)
Supplement: sj-docx-1-pus-10.1177_09636625221148697 – Supplemental material for Moving beyond narrow definitions of gene drive: Diverse perspectives and frames enable substantive dialogue among science and humanities teachers in the United States and United Kingdom [file sj-docx-1-pus-10.1177_09636625221148697.docx]

**SUPPLEMENTAL MATERIAL**

**Moving beyond narrow definitions of gene drive: Diverse perspectives and frames enable substantive dialogue among science and humanities teachers in the US and UK**

*Hartley, S.^1^, Stelmach, A.^1^, Delborne, J.A.^2^, Barnhill-Dilling, S.K.^2^*

*^1^University of Exeter Business School, University of Exeter, UK*

*^2^North Carolina State University*

**Table of Contents**

1. Table 1. Advance materials for participants
2. Selection of frames for focus groups discussions
3. Focus groups slides
4. References
5. **Table 1. Advance materials on gene drive for participants.**

| **Material type** | **Rationale** | **Reference** |
| --- | --- | --- |
| **Video**: ‘*What’s a gene drive?’* | To familiarise teachers with the concept and mechanisms of the gene drive technology | Risk Bites (2015) What’s a gene drive?  Available at:  https://tinyurl.com/2sxu9nrk  (accessed 10 November 2021) |
| **Video**: ‘*Gene editing can now change an entire species – forever’* | To provide teachers with an overview of current debates on the technology, including social and ethical issues | Kahn J (2016) Gene editing can now change an entire species – forever.  Available at:  https://tinyurl.com/5n87nta6  (accessed 10 November 2021) |
| **Open Letter**: ‘*A Call to Protect Food Systems from Genetic Extinction Technology’* | To familiarise teachers with the controversy surrounding gene drive and to present the point of view of the opponents of this technology. | ETC (n.d.) A Call to Protect Food Systems from Genetic Extinction Technology. Available at:  https://tinyurl.com/4nx365u4  (accessed 10 November 2021) |
| **Open Letter**: ‘*Open Letter: ‘Research on gene drive technology can benefit conservation and public health’* | To familiarise teachers with the controversy surrounding gene drive and to present the point of view of the supporters of this technology. | Target Malaria (2018) Open Letter: ‘Research on gene drive technology can benefit conservation and public health’. Available at:  https://tinyurl.com/ycktt2m7  (accessed 10 November 2021) |

1. **Selection of frames for focus groups discussions**

- **Stage 1**. In the first step we drew on data from our other projects on gene drive. We used data from our study of gene drive reporting in UK and US leading media (Stelmach et al., 2022), as well as from the interviews with stakeholders based in these countries (Russell et al., 2022). The second author compared both data sets and compiled a list of about 200 expressions used to describe various aspects of this technology. The criteria for selection at this stage was whether the linguistic expressions were frequent in the texts, whether they overlapped across the two sets of texts, or whether they were the focus of extended discussions during the interviews or in the media.
- **Stage 2**. References for all linguistic expressions were identified. Whenever an expression was sourced from interviews, additional searches were conducted to identify more sources of the expression, for example from the media, social science articles on gene drives, or popular websites. Expressions for which publicly available sources were not identified were excluded from the sample.
- **Stage 3**. We further narrowed the sample by assessing whether the linguistic expressions could be classed as examples of frames by using a typology of frames in science communication (Gamson and Modigliani, 1989; Nisbet, 2009).
- **Stage 4**. To ensure that the framings reflect both technical and social aspects of gene drive, as well as diverse perspective on this technology we grouped the framings into four categories: i) those explaining the mechanism and the scientific background of gene drive, ii) those describing possible applications, as well as ethical or societal implications of this technology, iii) those representing pro-gene drive perspective, iv) and those representing perspectives critical of gene drive. To determine the favourable/critical valence of these expressions, the research team discussed each of them, taking into account their use, the context in which they were deployed, and the attributable users of these expressions.
- **Stage 5**. In the final stage we chose 2 frames from each category. We limited our selection to 8 frames in total to make the discussion manageable. To maximise the discussion, we aimed to select a diverse range of framings, including those anchored in popular culture and evoking well-known books and films. In the selection process, we did not aim to achieve a statistically representative spread of gene drive framings, but rather sought to include a range of expressions most likely to stimulate debate.

**Table 2. Framings for focus groups discussions.**

| **Framing** | **Source** |
| --- | --- |
| Jurassic Park | Watts J (2018) GM mosquito trial sparks ‘Sorcerer’s Apprentice’ lab fears. *The Guardian*, 3 December. |
| Malaria-proof mosquito | Neergaard L (2016) Malaria-proof mosquito? Tool promising but needs more study. *St Louis Post-Dispatch*, 12 June. |
| GM on steroids | Avaaz (n.d.) Stop GM on steroids. Available at https://secure.avaaz.org/campaign/en/gm_on_steroids/ (accessed 4 November 2021). |
| Eradicate the world’s deadliest creature | Berezow A (2016) Mosquitoes, this time it’s war; Zika is the last straw: Eradicate the world’s deadliest creature. *USA Today*, 4 February. |
| Tweaking genes to save species | Rosner H (2016) Tweaking Genes to Save Species. *The New York Times*, 17 April. |
| Exterminator technology | Achenbach J (2018) ‘Genedrive’ research to fight diseases can proceed cautiously, U.N. group decides. *The Washington Post,* 2 December. |
| To bias inheritance | Kirkpatrick J and Light A (2015) The great potential – and great risks – of gene editing. *The Washington Post*, 12 December. |
| We don’t want to be guinea pigs | Pujol-Mazzini A (2019) ‘We don’t want to be guinea pigs’: how one African community is fighting genetically modified mosquitoes. *The Telegraph*, 8 October. |

1. **Focus groups slides**


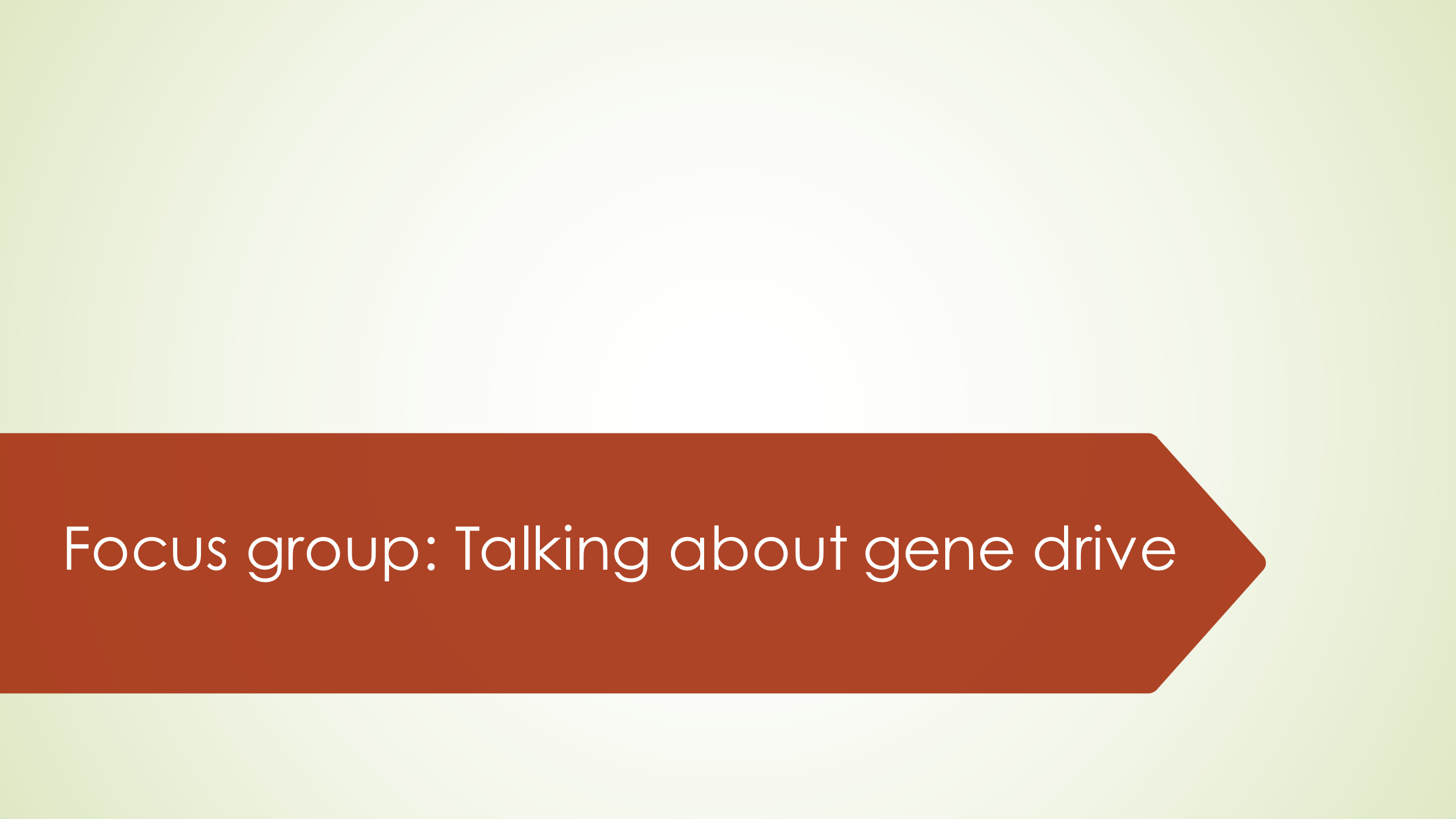

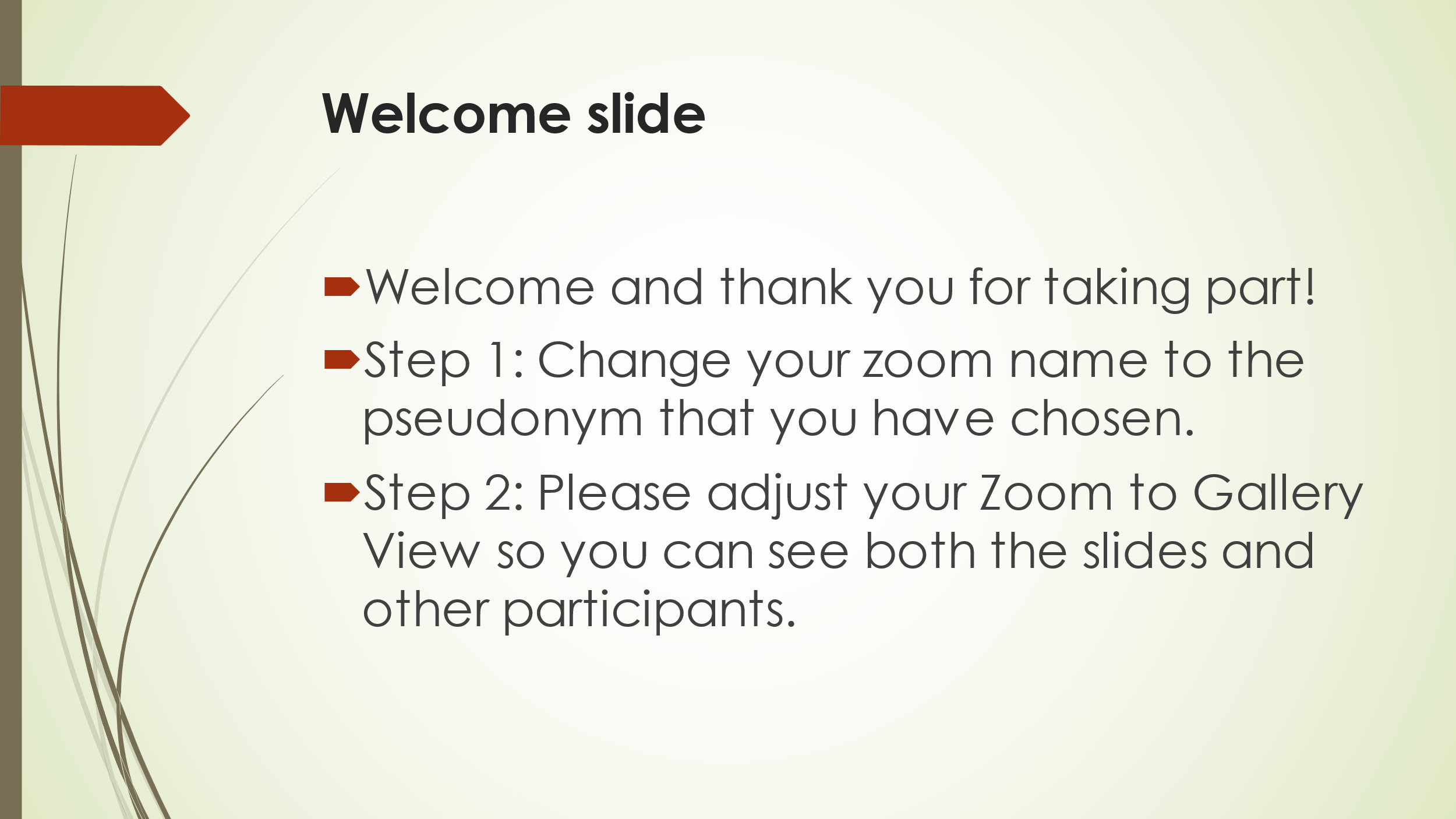

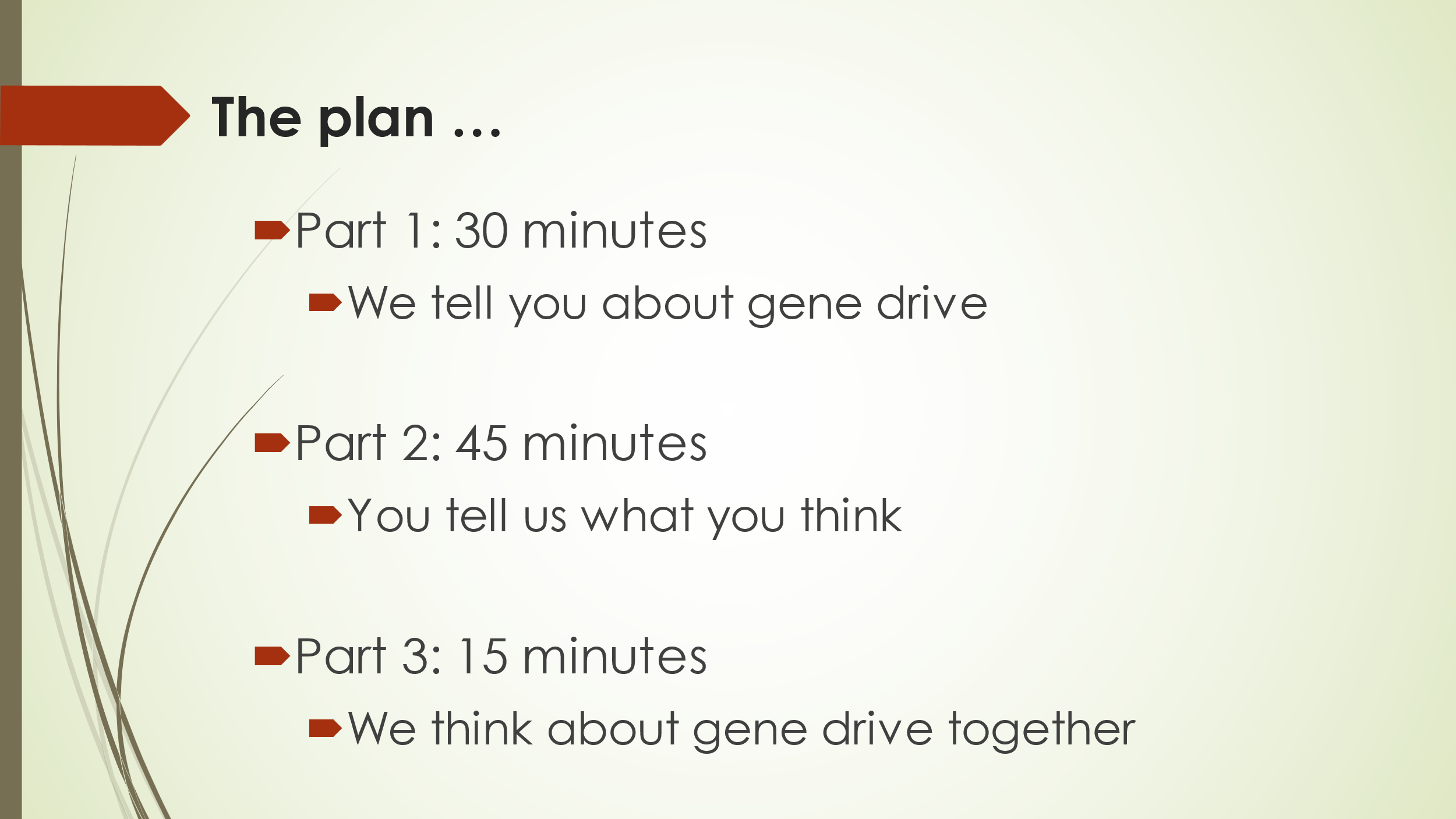

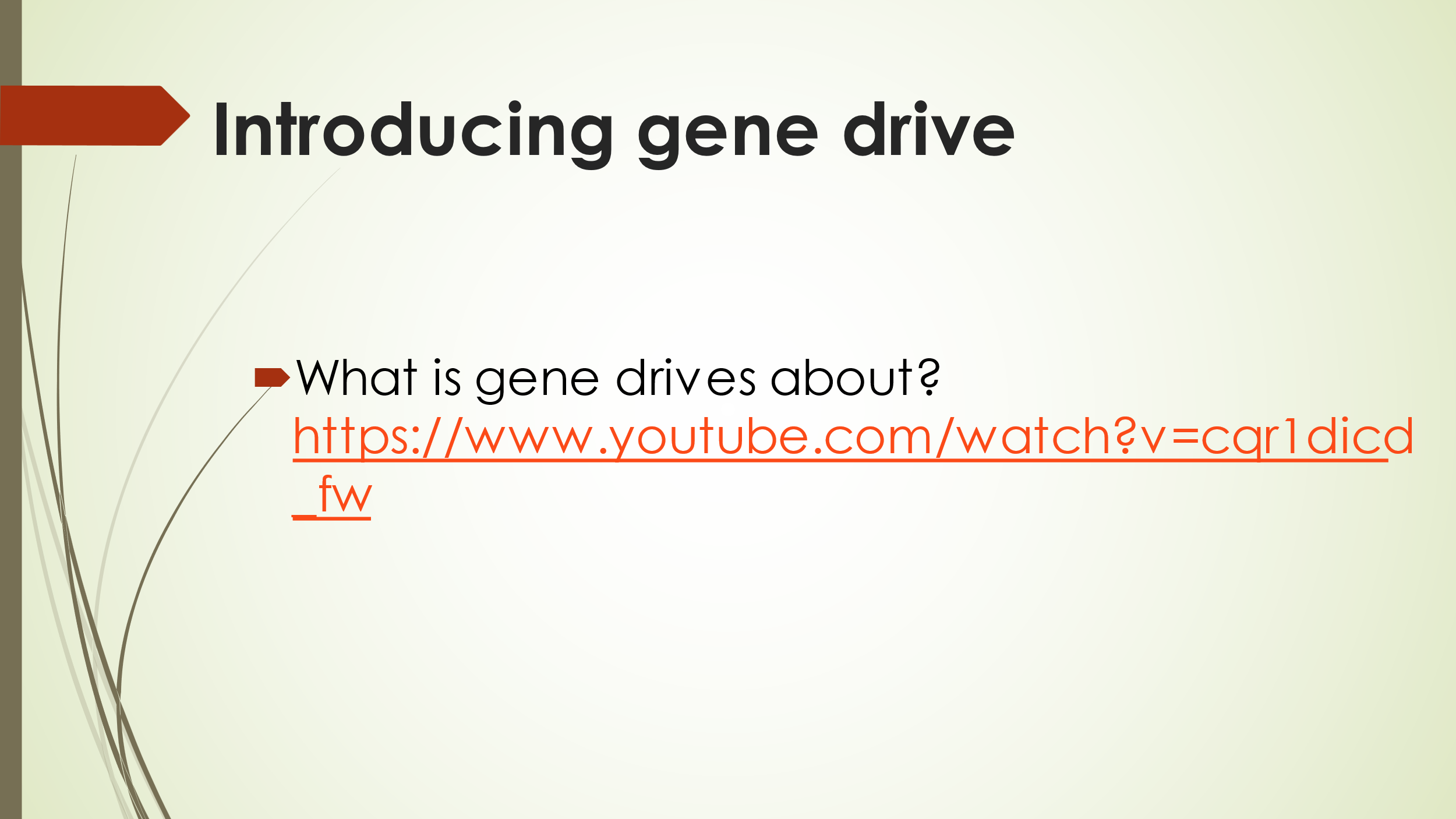

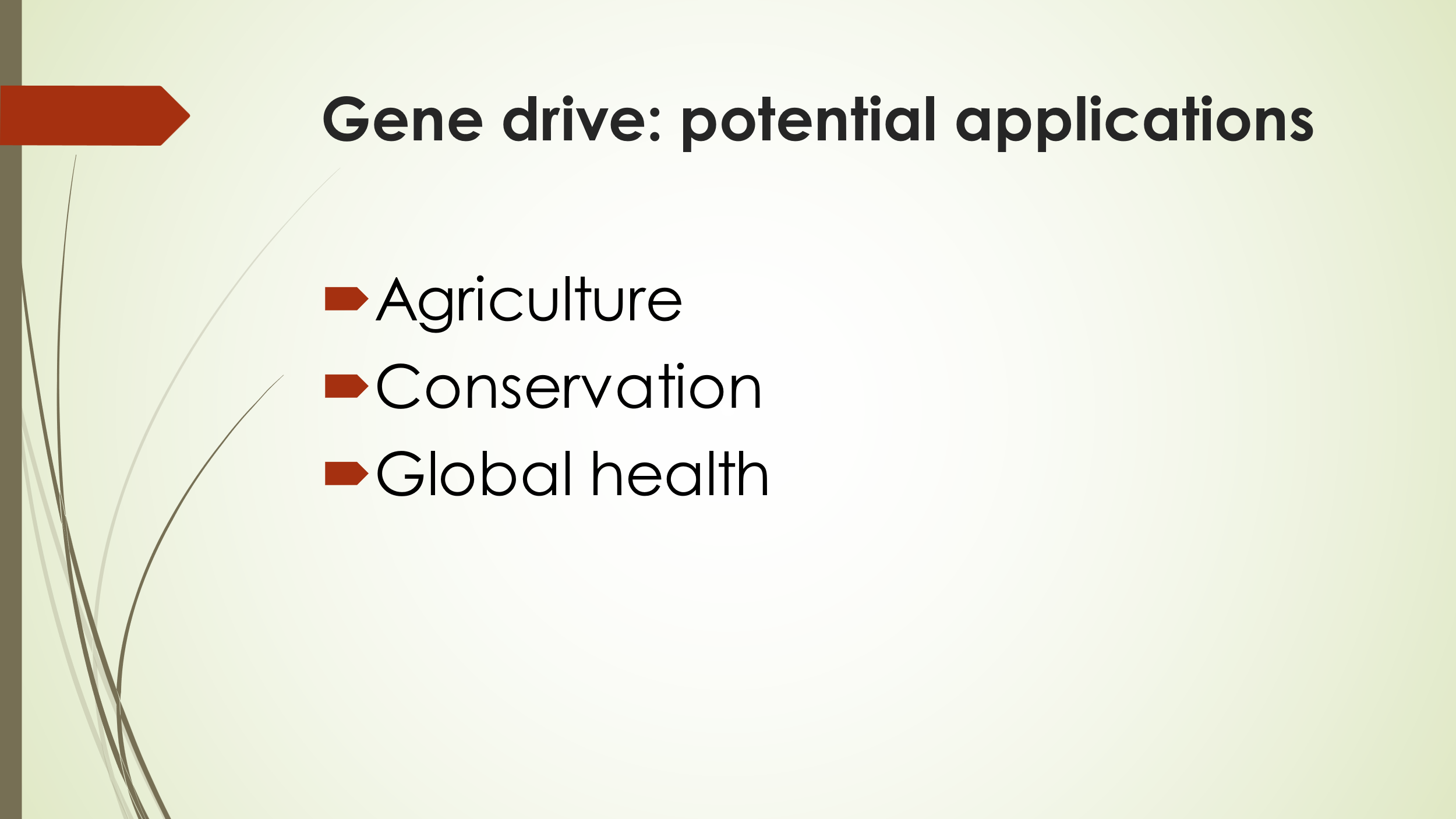

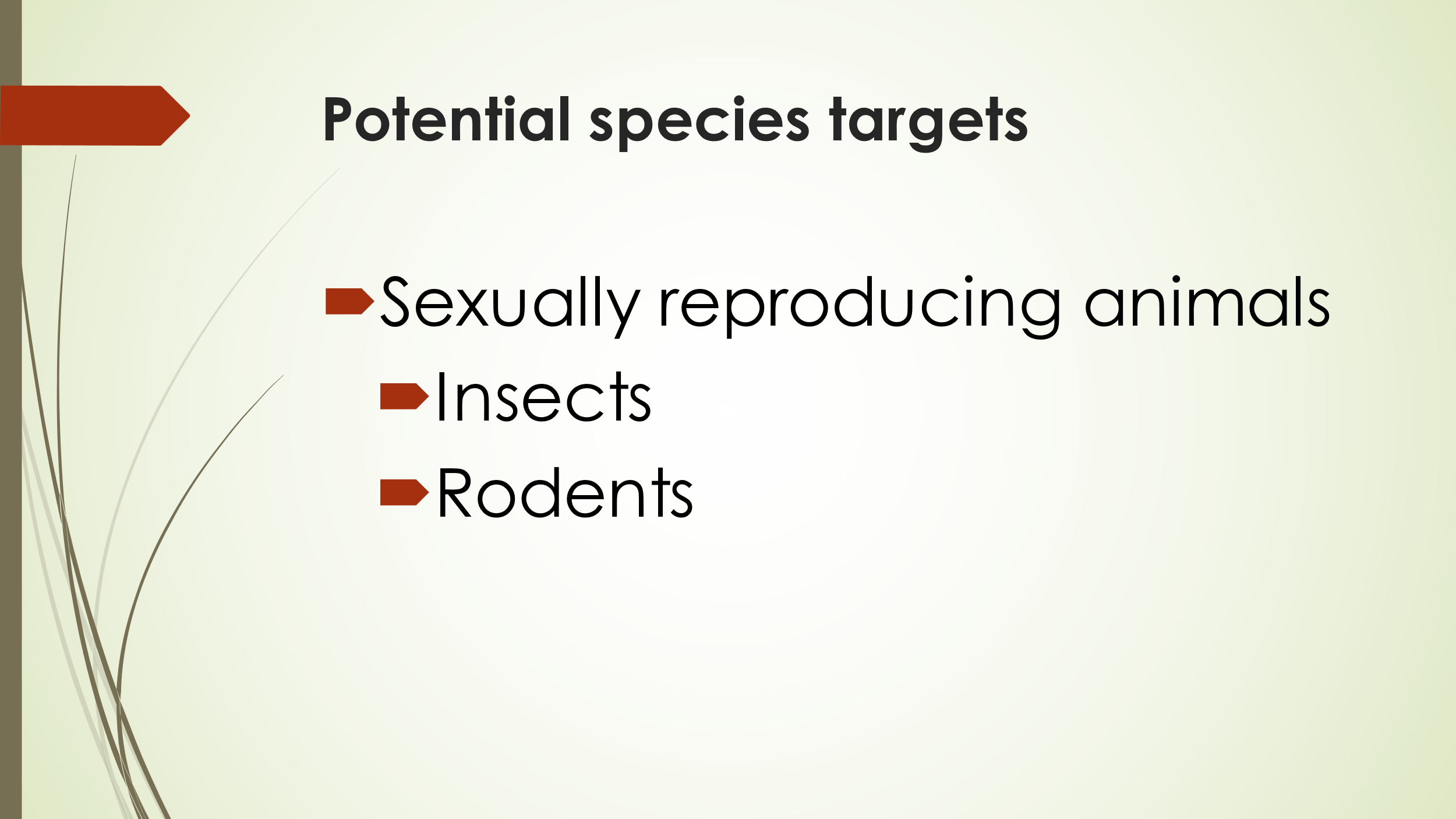

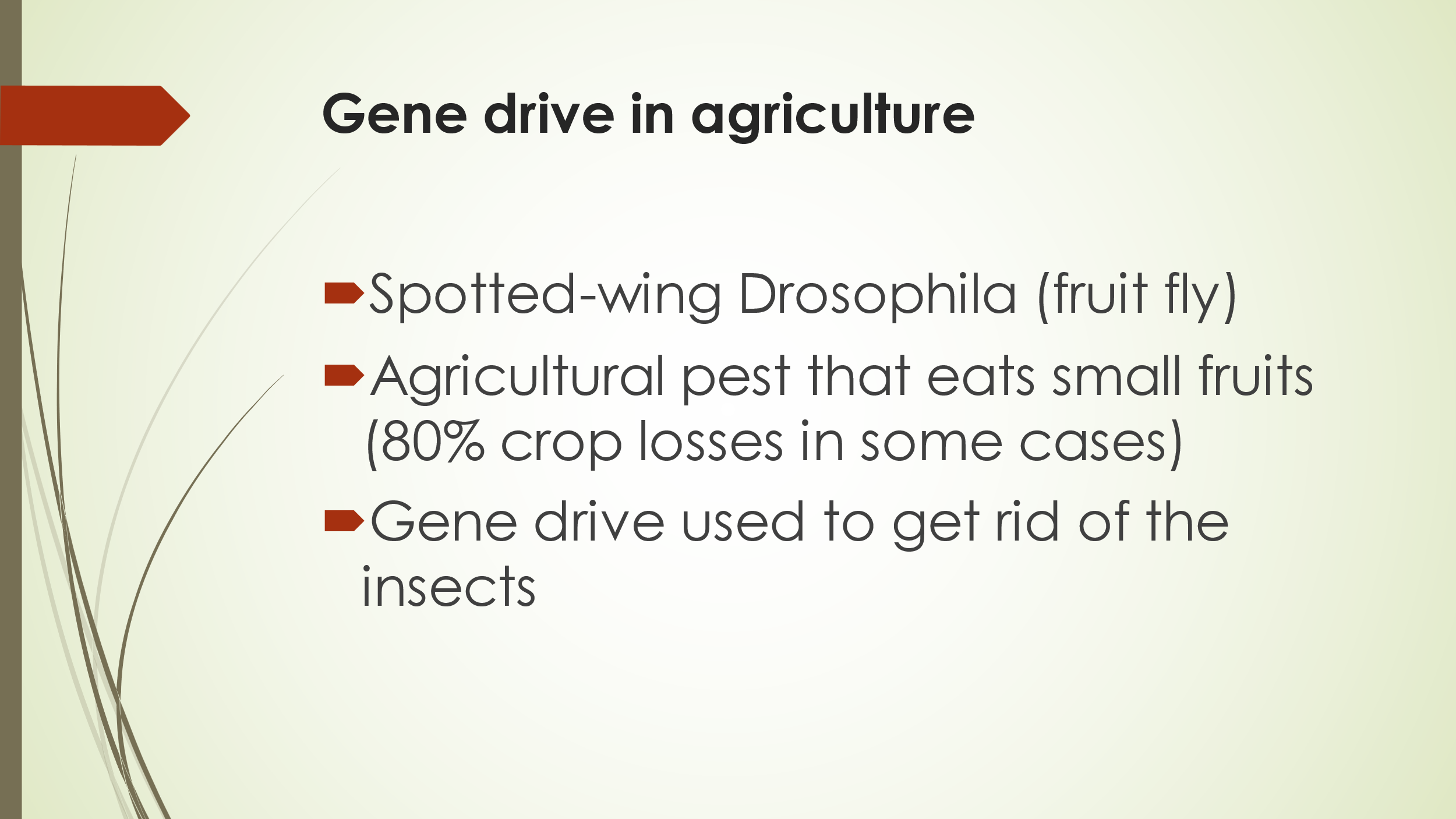

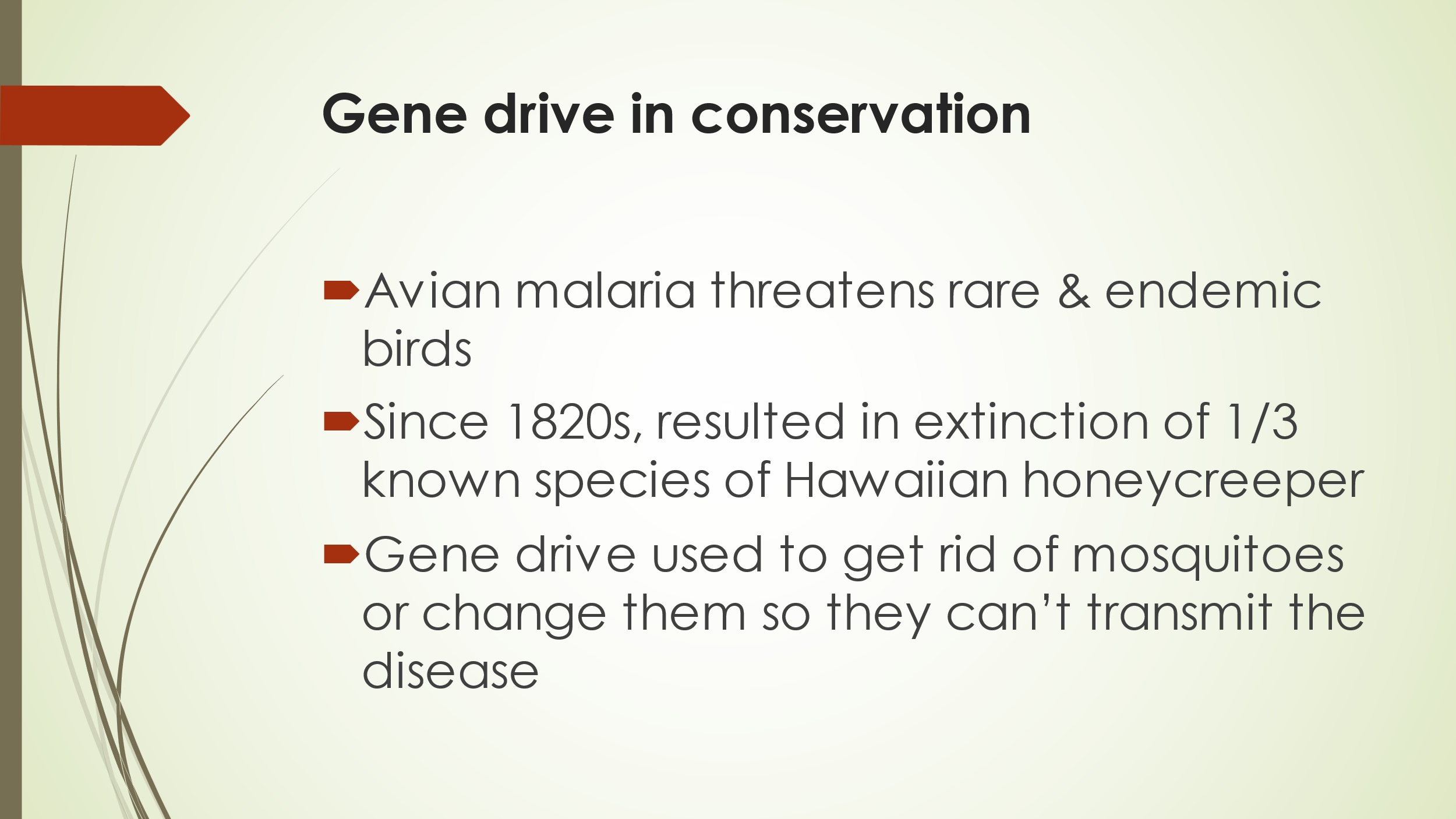

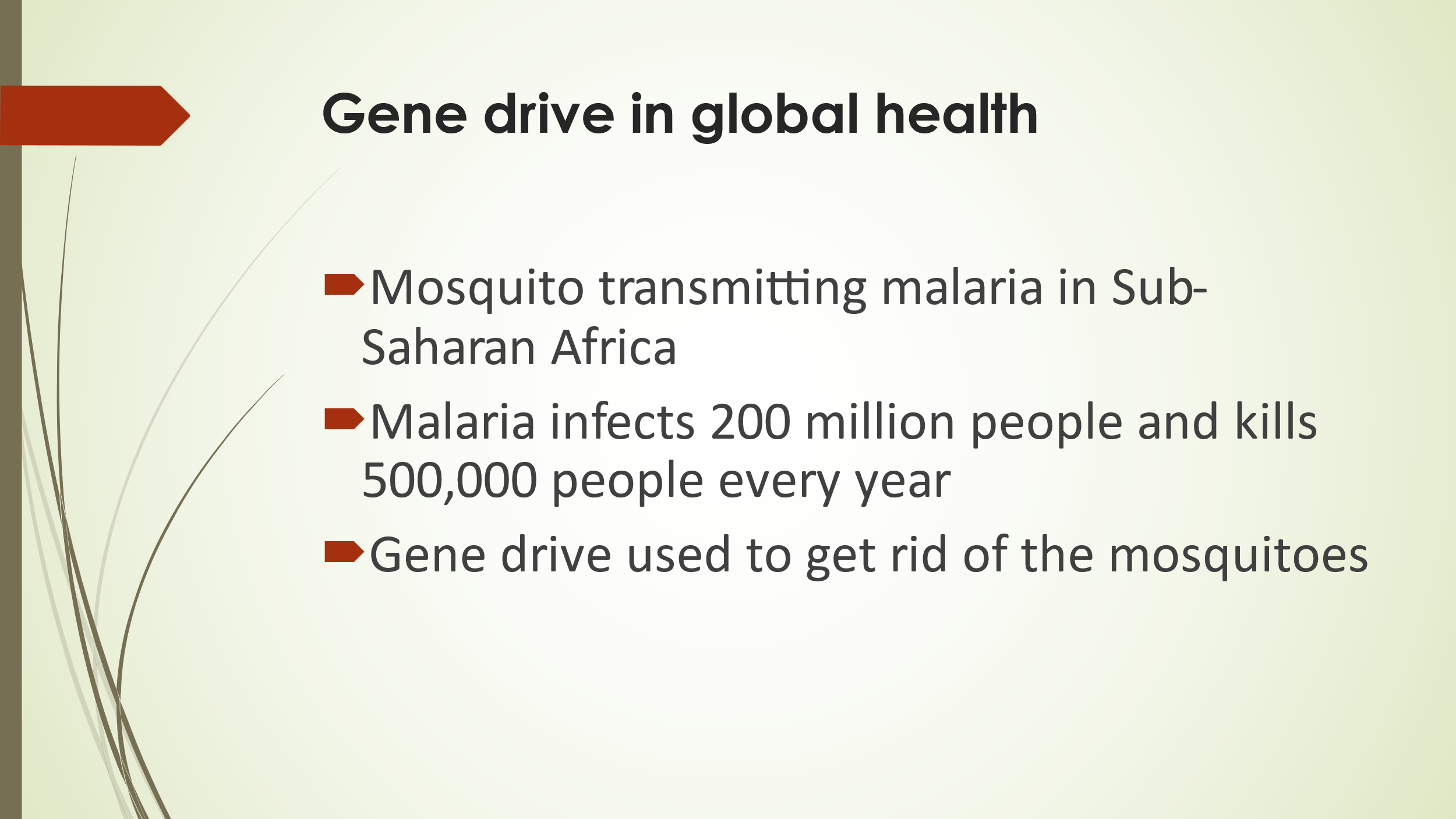

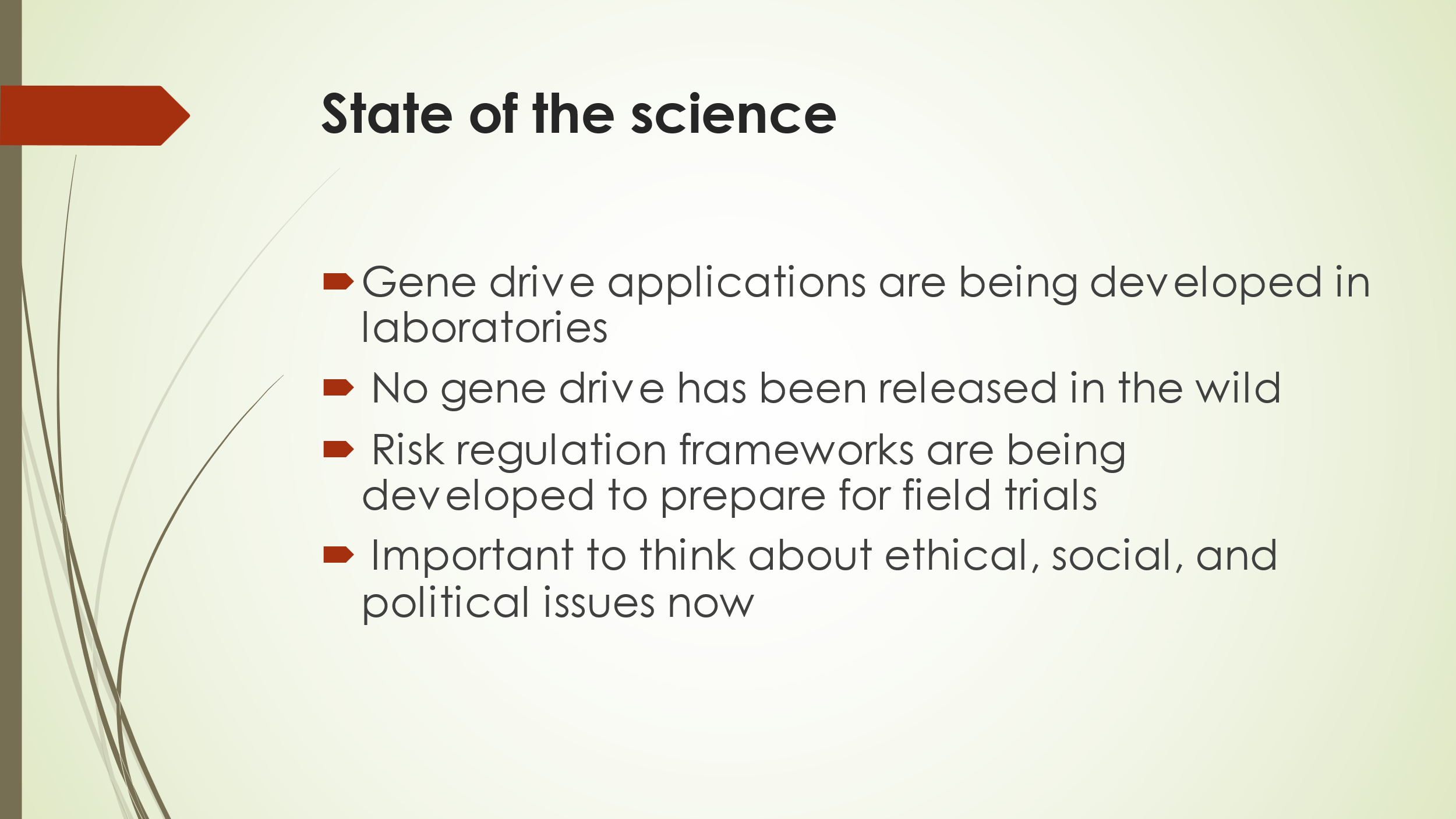

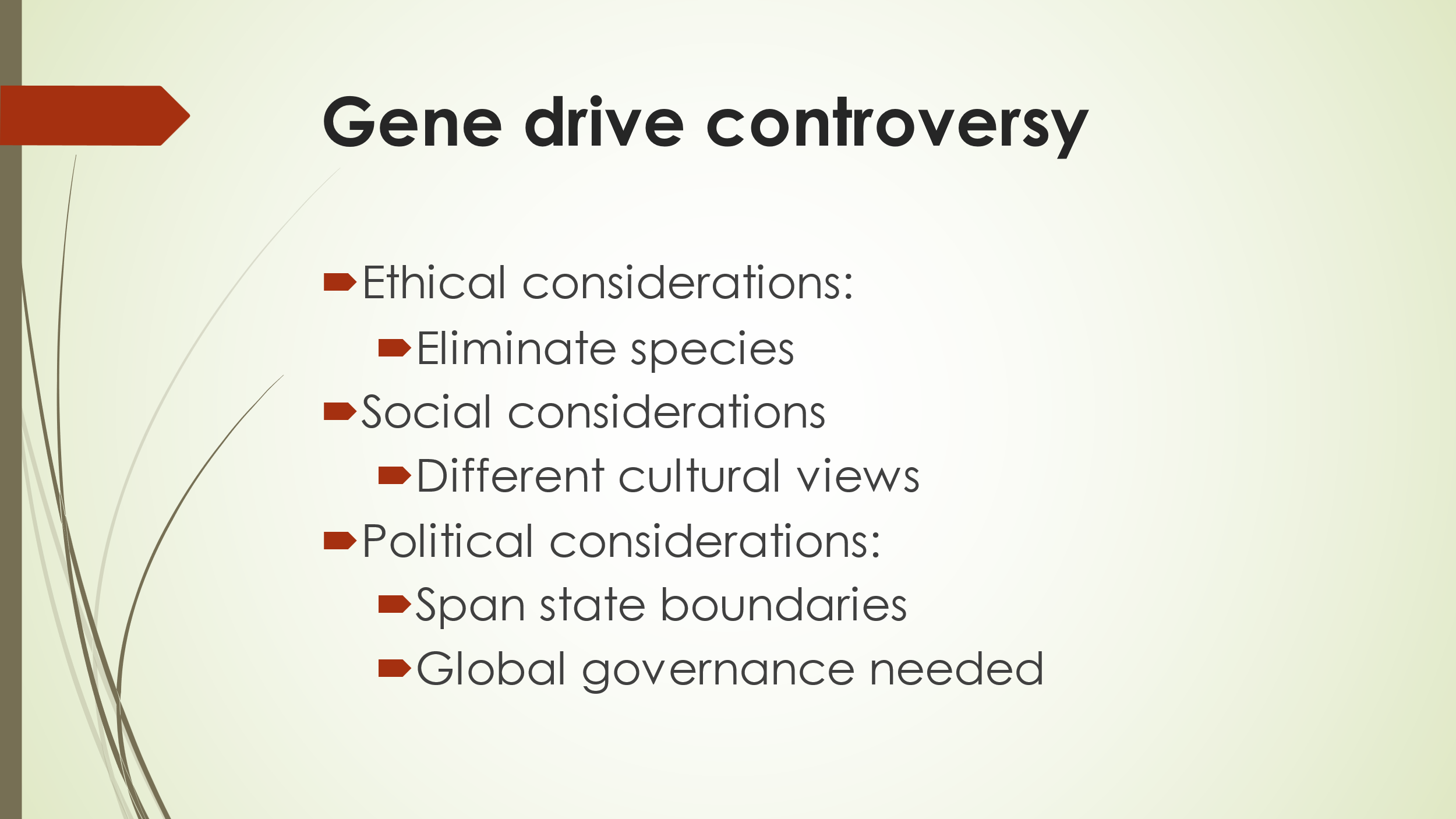

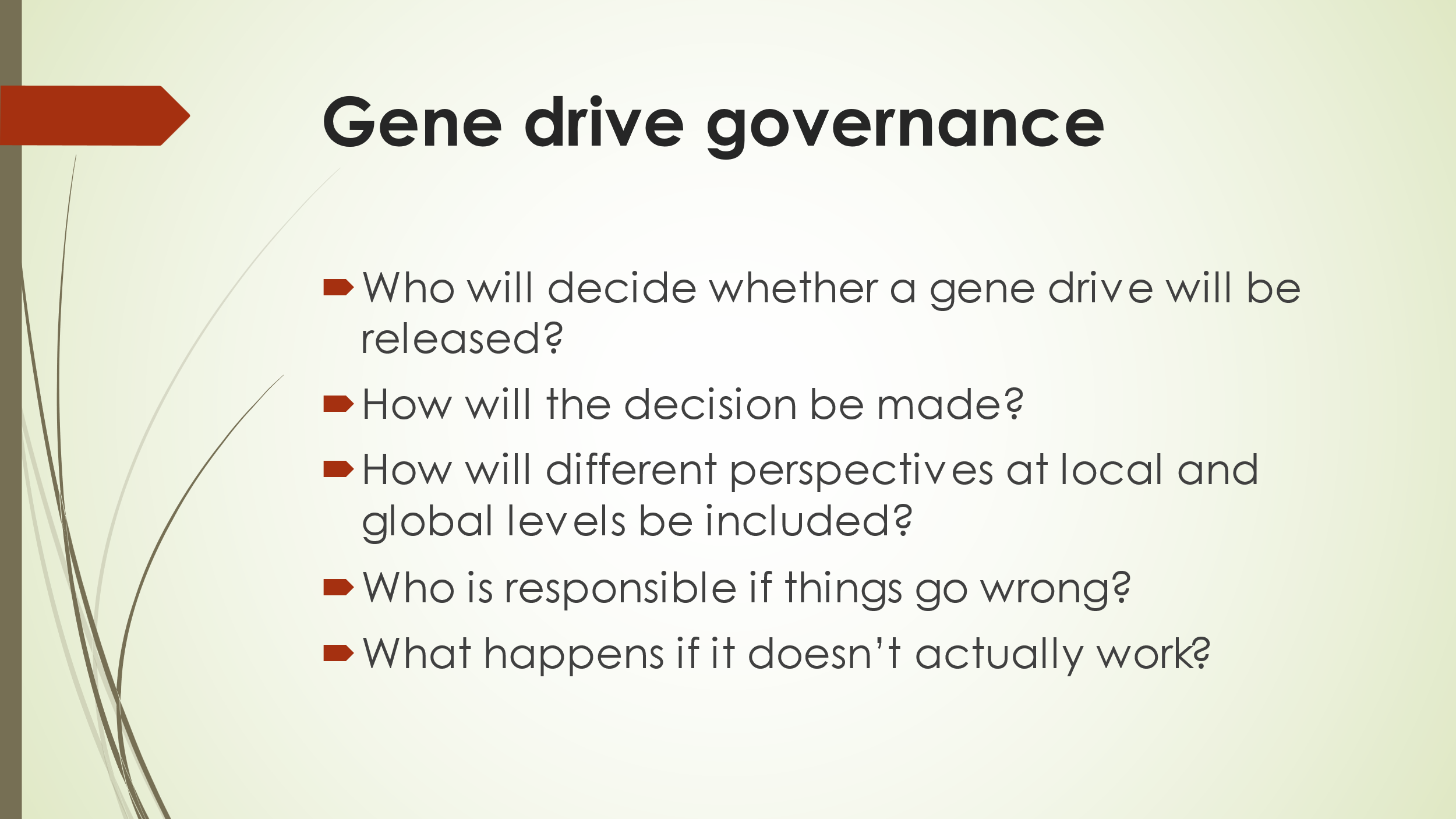

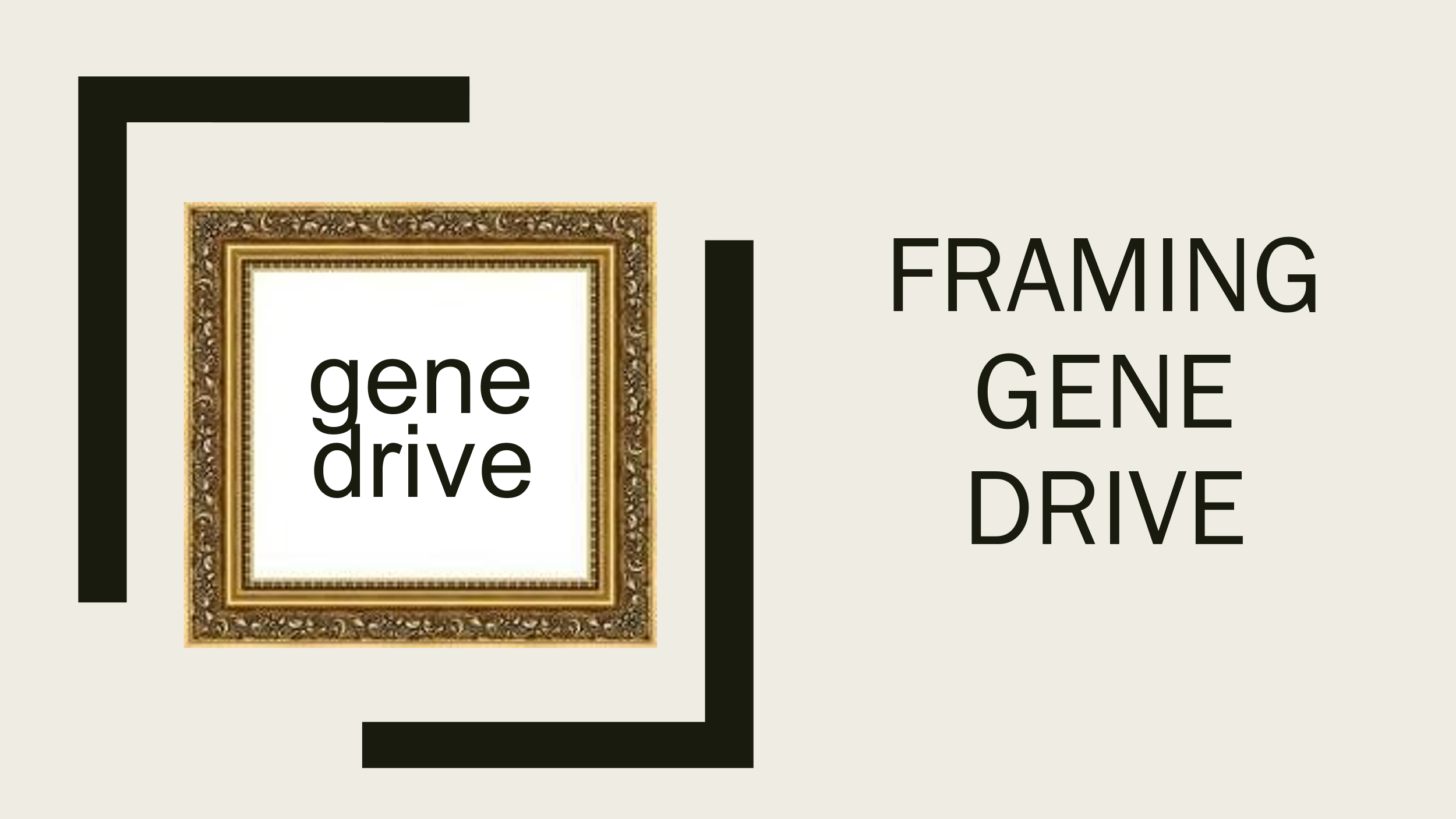

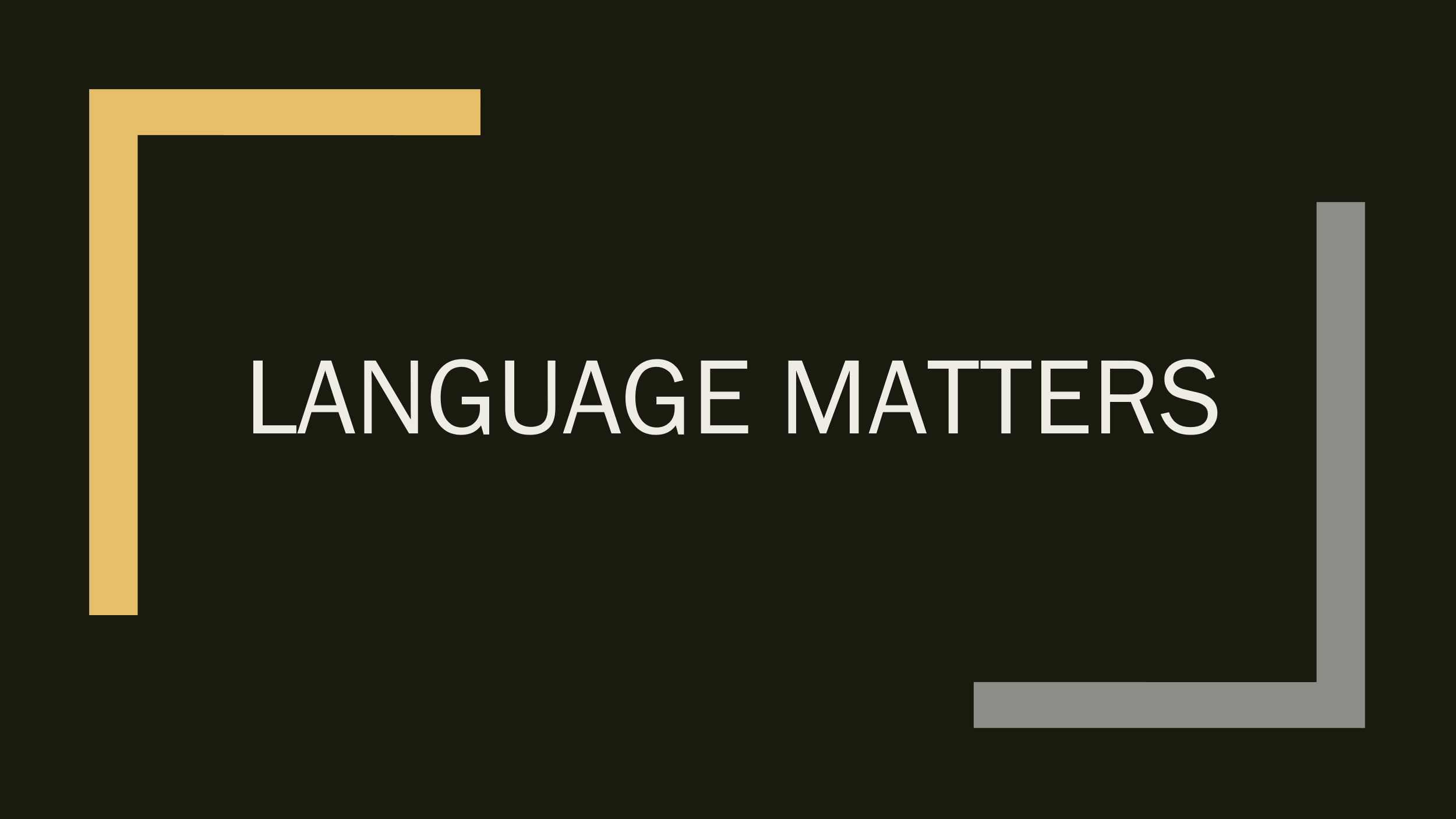

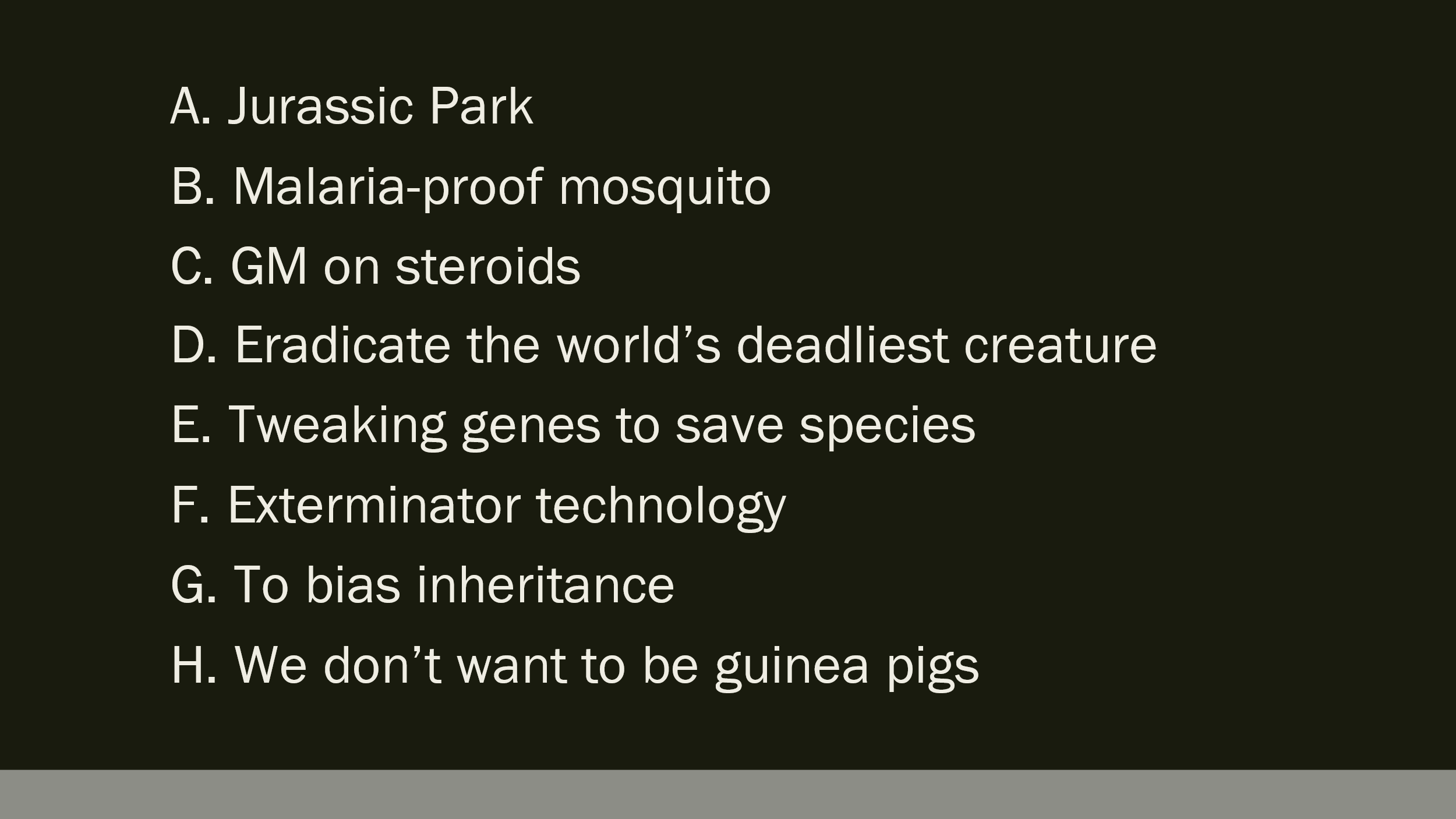

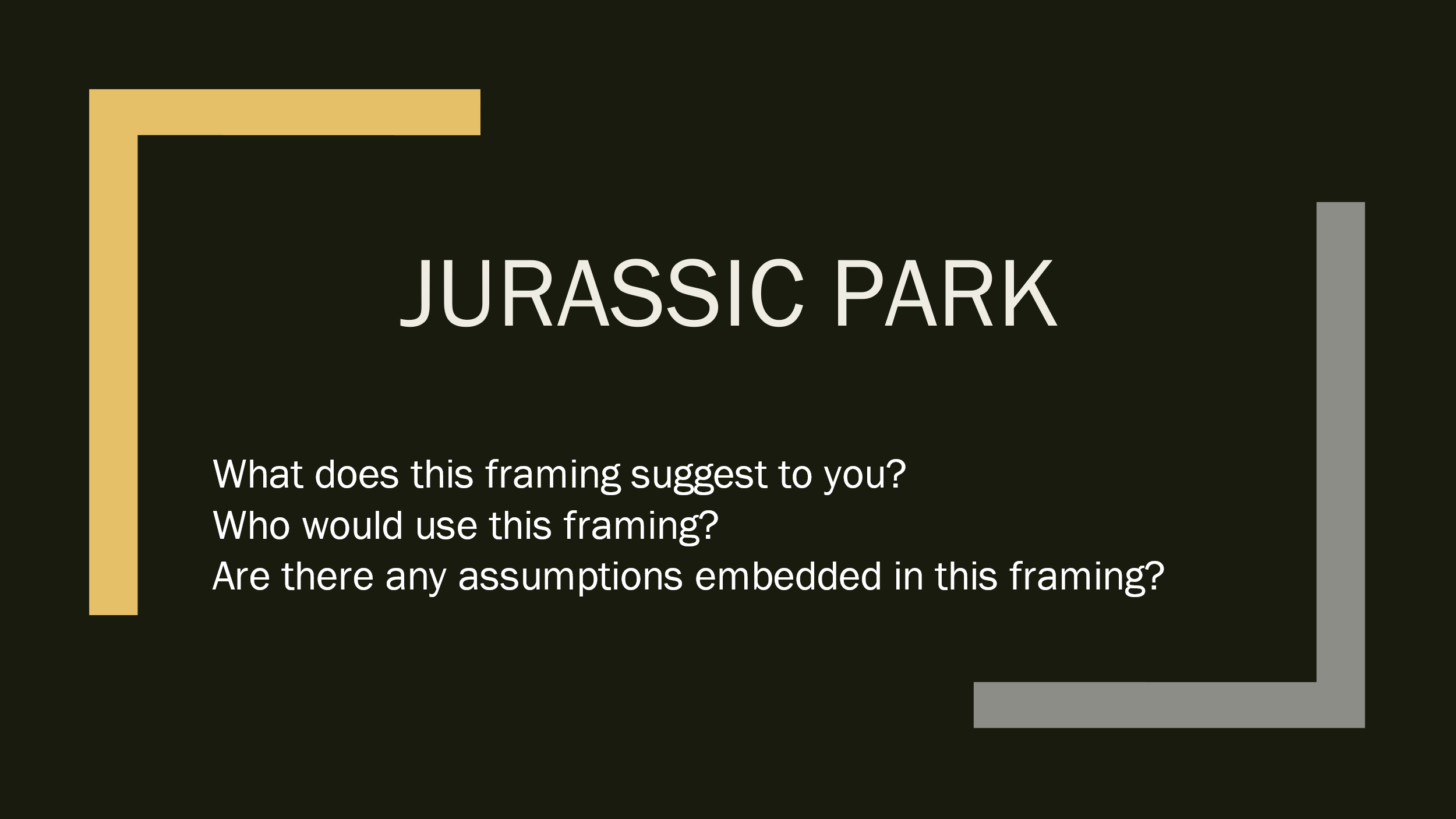

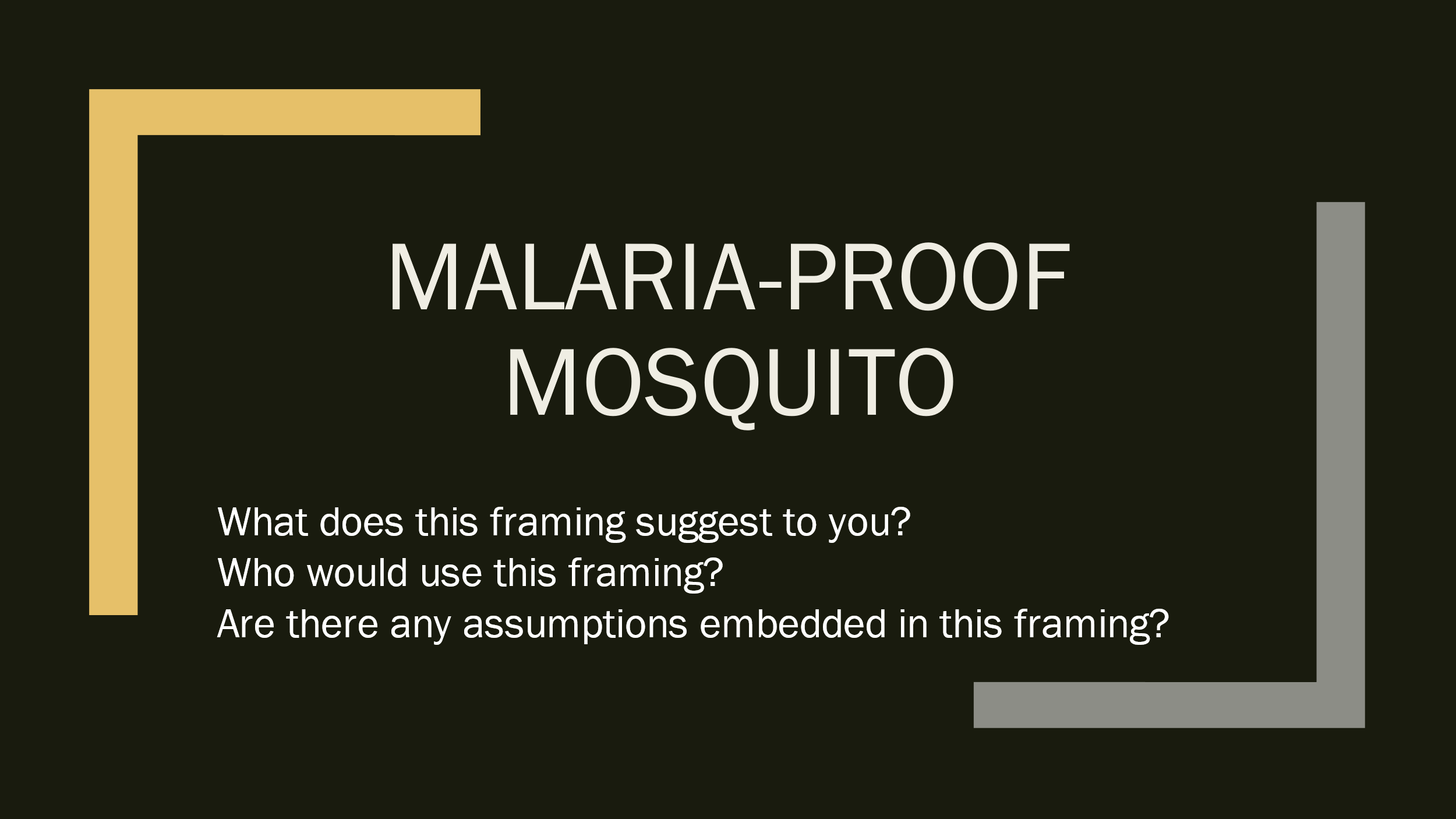

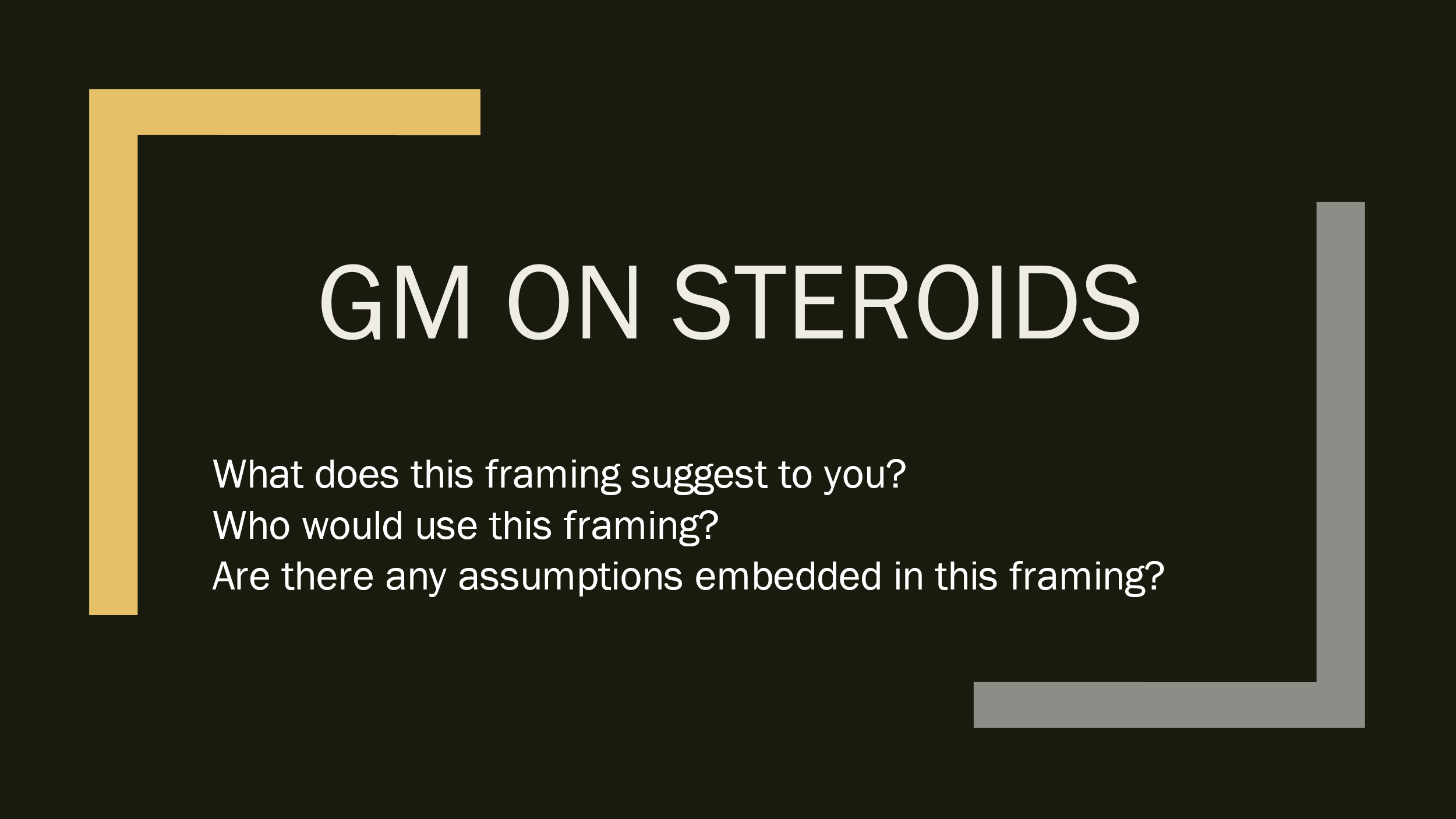

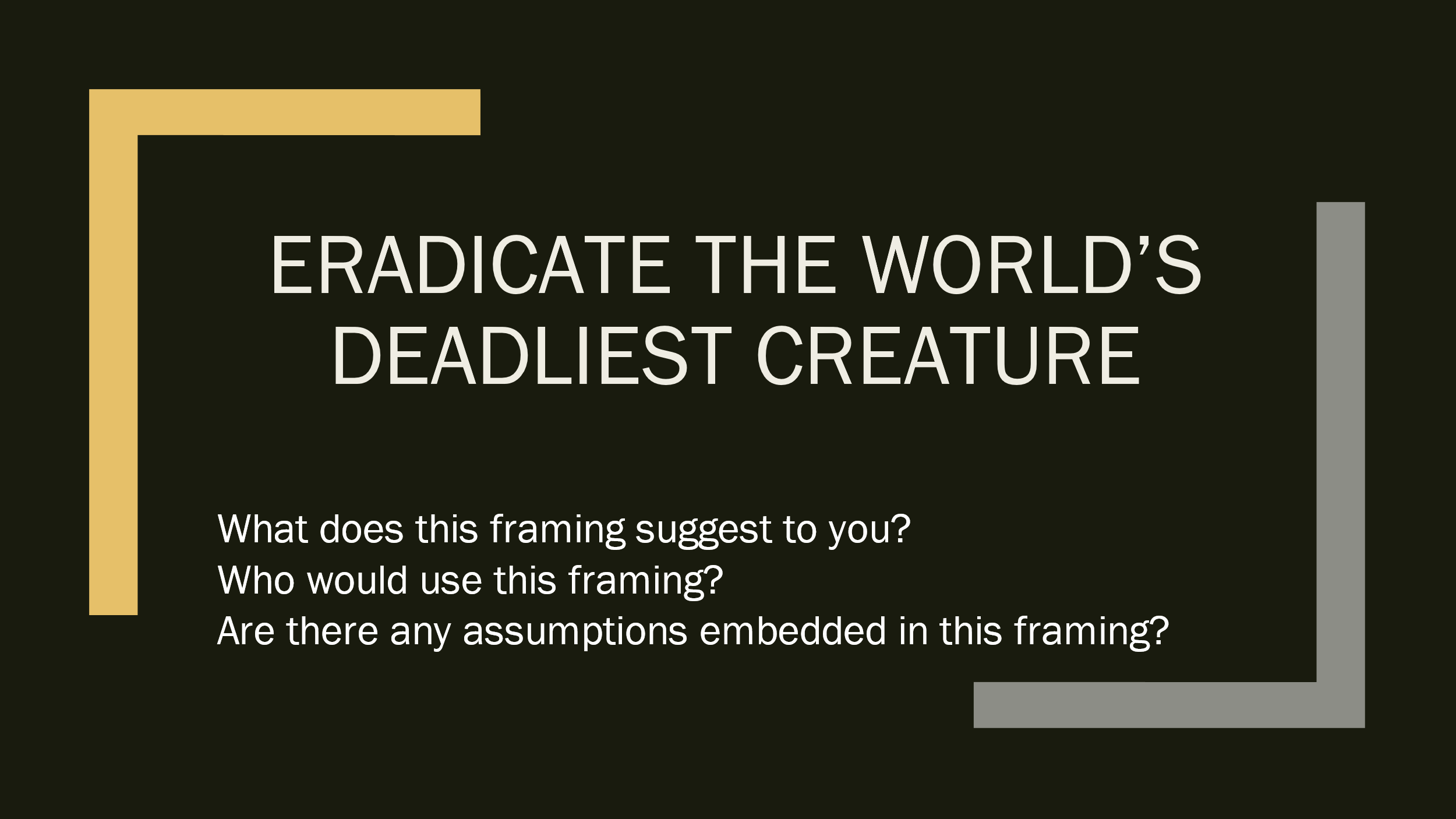

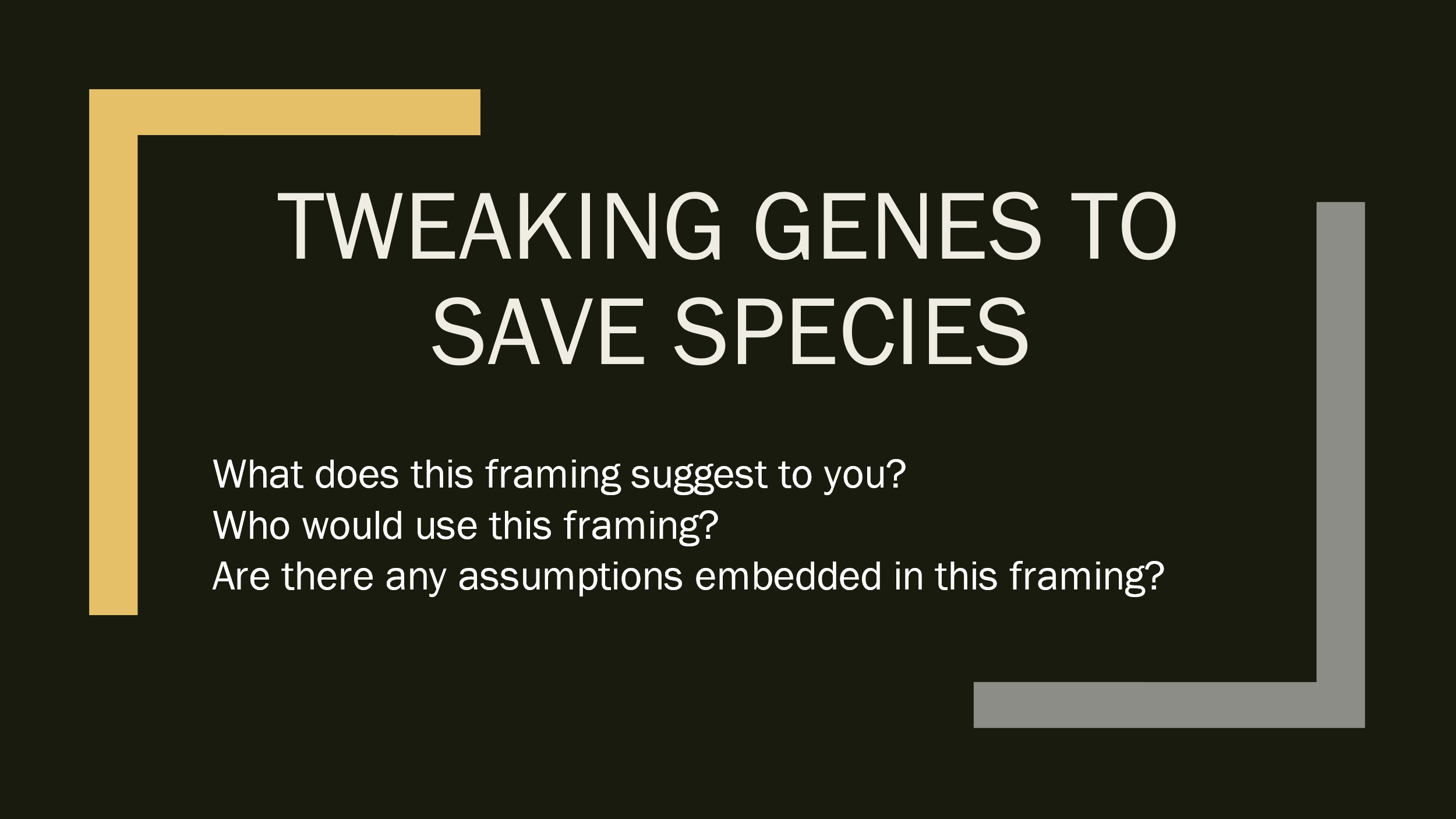

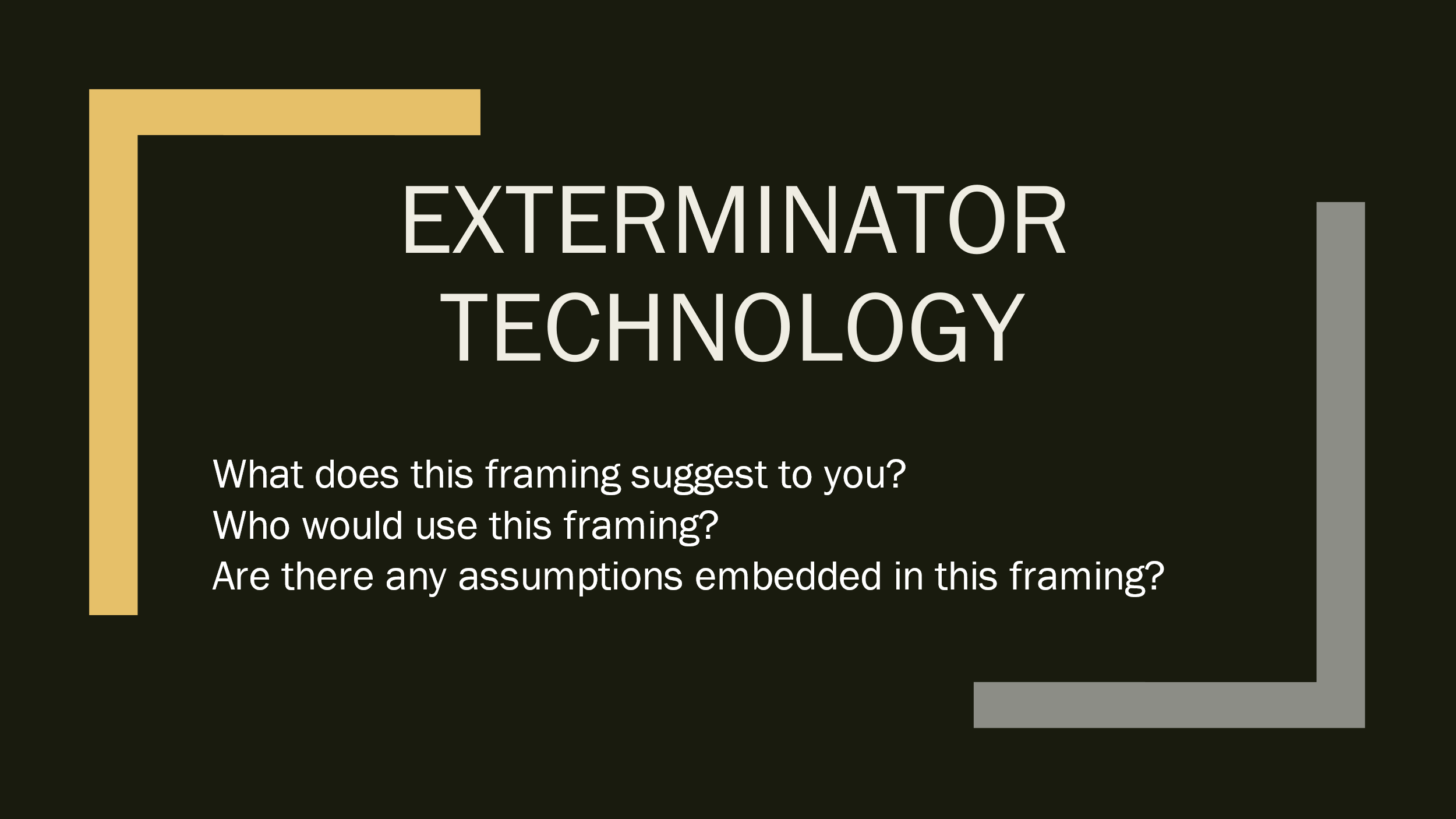

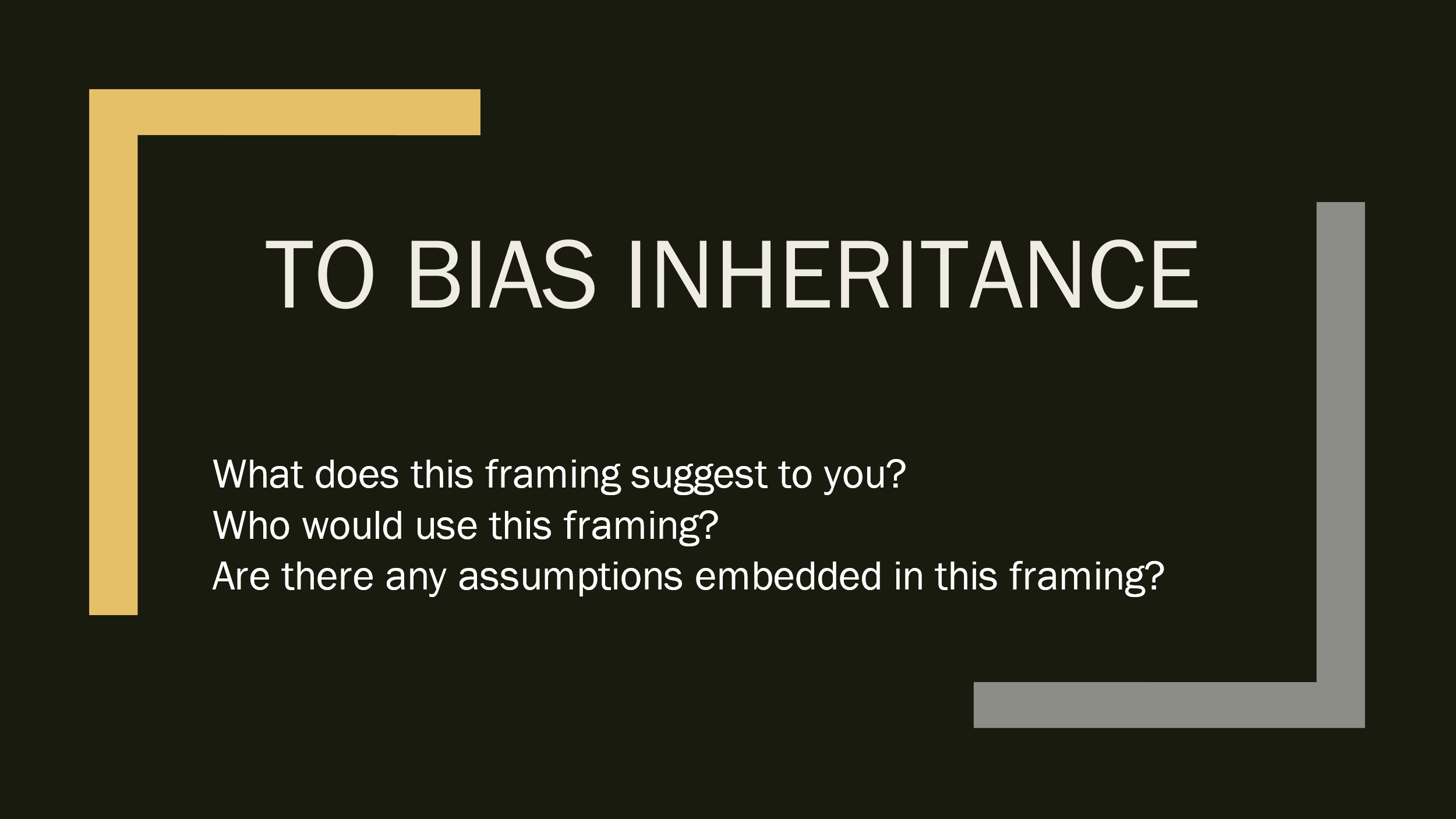

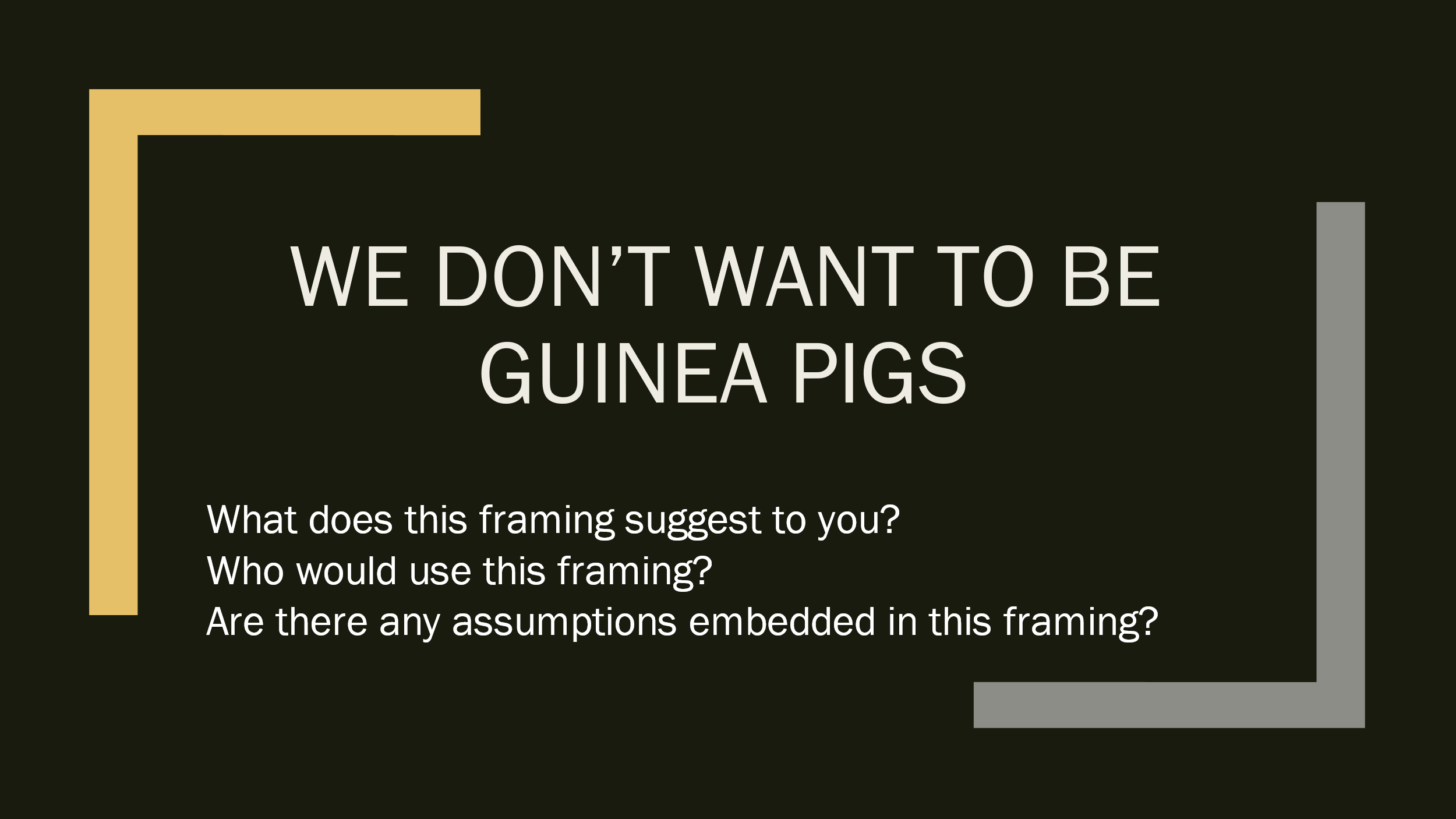

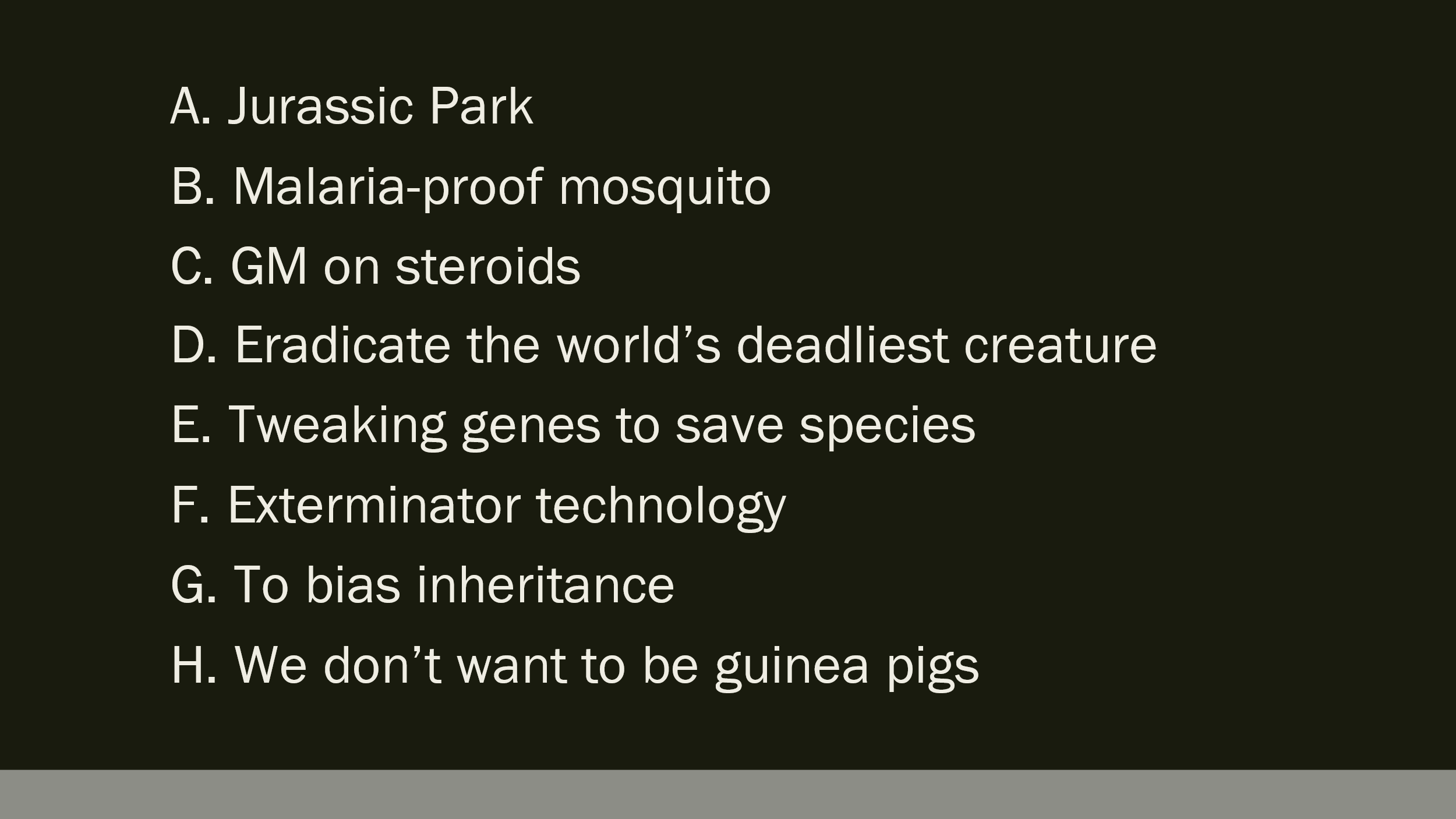

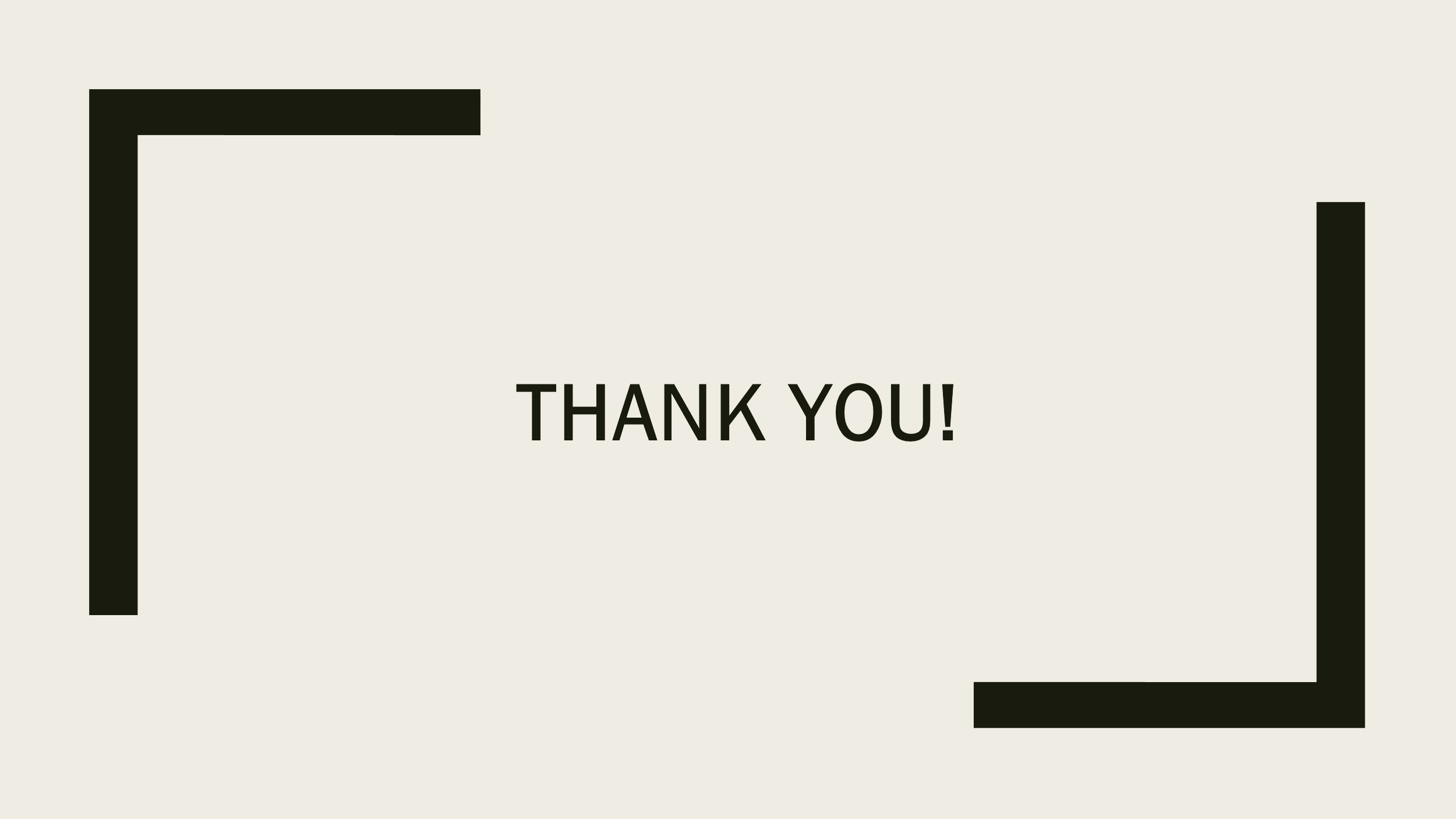


1. **References**

Gamson W A and Modigliani A (1989) Media discourse and public opinion on nuclear power: A constructionist approach. *American Journal of Sociology*, 95(1), 1-37.

Nisbet M C (2009) Framing science: A new paradigm in public engagement. In L. Kahlor and P. Stout (Eds.) *Communicating Science: New Agendas in Communication* (pp. 41-67). New York, NY: Routledge.
